# High-Density Linkage Map Construction and Mapping of Salt-Tolerant QTLs at Seedling Stage in Upland Cotton Using Genotyping by Sequencing (GBS)

**DOI:** 10.3390/ijms18122622

**Published:** 2017-12-05

**Authors:** Latyr Diouf, Zhaoe Pan, Shou-Pu He, Wen-Fang Gong, Yin Hua Jia, Richard Odongo Magwanga, Kimbembe Romesh Eric Romy, Harun or Rashid, Joy Nyangasi Kirungu, Xiongming Du

**Affiliations:** 1 State Key Laboratory of Cotton Biology/Institute of Cotton Research, Chinese Academy of Agricultural Sciences, Anyang 455000, China; bassf82@gmail.com (L.D.); panzhaoe@163.com (Z.P.); zephyr0911@126.com (S.-P.H.); gwf018@126.com (W.-F.G.); jiayinhua_0@sina.com (Y.H.J.); magwangarichard@yahoo.com (R.O.M.); harunbjri@yahoo.com (H.o.R.); nyangasijoy@yahoo.com (J.N.K.); 2 Senegalese River Valley Development Agency (SAED), Saint-Louis Bp74, Senegal; 3 School of Physical and Biological Sciences (SPBS), Jaramogi Oginga Odinga University of Science and Technology (JOOUST), Main Campus, P.O. Box 210-40601 Bondo, Kenya; 4 Chinese National Rice Research Institute (CNRRI), Chinese Academy of Agricultural Sciences, Hangzhou 311400, China; thekingmbembe@gmail.com

**Keywords:** upland cotton, high-density, salinity tolerance, QTL mapping, GBS

## Abstract

Over 6% of agricultural land is affected by salinity. It is becoming obligatory to use saline soils, so growing salt-tolerant plants is a priority. To gain an understanding of the genetic basis of upland cotton tolerance to salinity at seedling stage, an intra-specific cross was developed from CCRI35, tolerant to salinity, as female with Nan Dan (NH), sensitive to salinity, as the male. A genetic map of 5178 SNP markers was developed from 277 F_2:3_ populations. The map spanned 4768.098 cM, with an average distance of 0.92 cM. A total of 66 QTLs for 10 traits related to salinity were detected in three environments (0, 110, and 150 mM salt treatment). Only 14 QTLs were consistent, accounting for 2.72% to 9.87% of phenotypic variation. Parental contributions were found to be in the ratio of 3:1, 10 QTLs from the sensitive and four QTLs from the resistant parent. Five QTLs were located in A_t_ and nine QTLs in the D_t_ sub-genome. Moreover, eight clusters were identified, in which 12 putative key genes were found to be related to salinity. The GBS-SNPs-based genetic map developed is the first high-density genetic map that has the potential to provide deeper insights into upland cotton salinity tolerance. The 12 key genes found in this study could be used for QTL fine mapping and cloning for further studies.

## 1. Introduction

Currently, it is estimated that over 6% of agricultural land is affected by salinity [1]. To satisfy the increasing population demand, it is becoming obligatory to use saline soils, either by reclamation or by growing salt-tolerant plants [2]. To mitigate the challenge of salt stress, selective breeding programs are adopted in order to improve the performance of the genotypes developed [3]. However, the majority of traits with economic significance, such as salinity tolerance, are regulated by quantitative trait loci (QTL) and thus are environment-dependent [4]. Using conventional breeding techniques to enhance genetic improvement has limitations due to low efficiency, slow speed, and being expensive for some traits like salt tolerance [4]. Molecular breeding, such as marker-assisted breeding (MAS) and genomic selection (GS), have the potential to overcome the inefficiencies of conventional breeding [5]. The most valuable tool in molecular breeding is genotype by sequencing (GBS) [6]. Molecular breeding techniques such as GBS have the potential to bridge the genotyping gap between references of broad interest, mapping, and/or breeding populations of specific interest [7]. The analysis of genotypic samples through genotyping by sequence (GBS) lowers the cost of molecular work, even though the resultant next-generation sequencing data have immediate applications in many different research areas, ranging from gene discovery to genomic-assisted breeding [7]. Producing a large amount of unbiased markers in an inexpensive way makes GBS the most preferable approach to building high-density and high-resolution maps, genomic selection, and facilitating QTL mapping [8].

QTLs mapping has become an important tool for quantitative trait research, and has been widely used in the agricultural field to map a number of traits including salt tolerance in various crops [9]. A number of QTLs linked to salinity tolerance traits have been identified in plants such as Arabidopsis [10], rice, [11], barley [12], and tomatoes [13]. In upland cotton, little research has been done towards determining the effect of salt stress on cotton at the seedling stage. However, previous studies reported an association mapping study for salinity stress tolerance in cotton [14], differentially expressed genes, and transcriptional regulation induced by salt stress in two contrasting cotton genotypes [15]. Many genes controlling response to high salinity have been identified in model plants, but only a few salt stress-inducible genes have been documented, such as ERF(*GhERF2-GhERF6*) [16,17], CCCH-type zinc finger (*GhZFP1*) [18], NHX1 (*GhNHX1*) [19], NAC (*GhNAC1-GhNAC6*) [20], metallothionein (*GhMT3a*) [21], GhMPK2 [22], GhMKK1 [23], and DREB (*GhDREB1*) [24]. However, to date, according to our best knowledge, only one report on QTL mapping for salt tolerance at seedling stage in the inter-specific cross of *Gossypium tomentosum* with *Gossypium hirsutum* has been reported [25]. It focused on semi-wild plants and morphological QTLs mapping traits.

According to previous research [26], the seedling stage of cotton is the most sensitive to salinity and the effects can be quantified by measuring morphological, physiological, and biochemical traits [27]. Identifying proper selection criteria for salinity tolerance is a major issue in breeding programs. The key point of our experiment was to simulate field conditions by sowing the seeds in different salt concentrations to investigate the response of seedlings to salt stress. Besides measuring parameters such as Malondialdehyde (MDA), Chlorophyll Content (CHL), and Electric Conductivity (EC) that act on biochemical and physiological levels, a comparison of determinant traits such as germination and growth parameters of genotypes in salinity was also undertaken.

In this study, a genetic map consisting of 5178 filtered GBS markers was developed using 277 F_2:3_ populations derived from an intra-specific cross between two upland cotton accessions, mainly cultivated in China, CCRI35 (tolerant to salinity) as the female parent and Nan Dan Ba Di Da Hua (NH) (sensitive to salinity) as the male parent. The genetic map was used to analyze QTLs associated with salt-tolerant traits using QTL cartographer [28]. The purpose of the study was to map the QTLs for salt tolerance and identify the candidate genes underlying the QTLs and their position in the cotton genome at the seedling stage. The findings of this study will provide valuable insights for breeders looking to develop salt-tolerant cultivars and enhance selection.

## 2. Results

### 2.1. Quality Control and Mapping to Reference Genome

The parental lines CCRI35 and NH were sequenced using the GBS method with efficient sequencing depths. In regard to CCRI35 and NH, an average of 10 individual reads for each of the parents were mapped to the sequence of the cotton genome (http://mascotton.njau.edu.cn) and totaled 13,695,154 and 13,496,550, respectively. A total average of 85,372 and 117,128 SNPs were identified in CCRI35 and NH, respectively. The efficiency of enzyme digestion was 99% for both of the parental lines. For the F_2:3_ populations, the efficiency of enzyme digestion was slightly low, 98.85%, compared to the parental lines. A total of 1,507,193,217 mapped reads were produced, with an average of 5,074,724.636 mapped reads per individual, which corresponds to nearly 186.98 Gb of clean base. This is equivalent to approximately 83.13-foldhaploid genome coverage of raw paired-end Illumina reads by sequencing whole-genome shotgun (WGS) libraries of homozygous cv. “TM-1” compared to Li Fuguang et al. (2015) [29] in their study, which generated a total of 445.7 Gb of clean reads, or 181-fold haploid genome coverage, of raw paired-end Illumina reads by sequencing whole-genome shotgun (WGS) libraries of homozygous cv. “TM-1” had fragment lengths ranging from 250 bp to 40 kb. The average GC content of the sequences is 38.25%, with a Q20 score of 94.66%.

### 2.2. SNP Detection and Annotation

The parents, CCRI35 and NH, were heterozygous and homozygous lines with AC and AA genotypes, respectively. The genotype AC × AA, consisting of 93,384 markers, was used for further analysis. Among the 93,384 markers, the low-coverage sequences of the F_2:3_ populations (coverage less than 75%) were filtered out, leaving 24,549 markers. Markers with significant distortion (*p* < 0.001) were filtered out and 6405 markers were retained with the purpose of determining bin markers.

### 2.3. Phenotypic Variation between Parents

**Figure 1:**
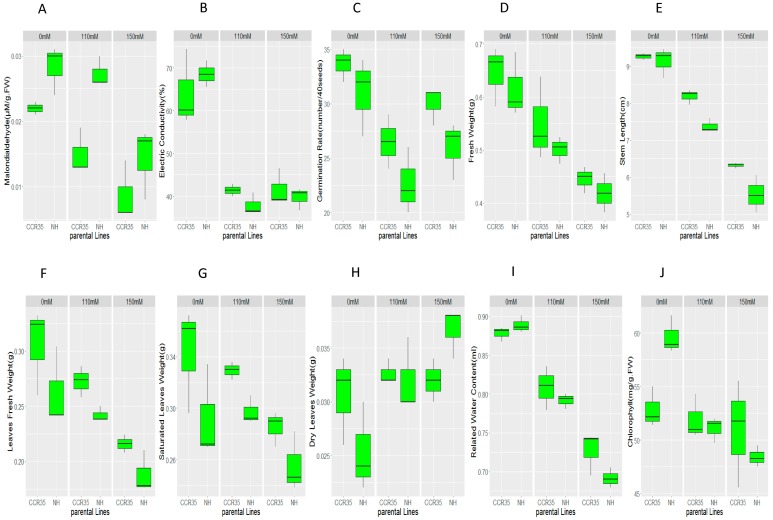
Phenotypic analysis of salt tolerance at the seedling stage of the two parents; (**A**) MDA: Malondialdehyde (µM/g.FW); (**B**) EC: Electric Conductivity (%); (**C**) GR: Germination Rate (number/40 seeds); (**D**) FW: Fresh Weight (g); (**E**) SL: Stem Length (cm); (**F**) LFW: Leaves Fresh Weight (g); (**G**) SLW: Saturated Leaves Weight (g); (**H**) DLW: Dry Leaves Weight (g); (**I**) RWC: Related Water Content (mL) and (**J**) CHL: Chlorophyll Content (mg/g.FW).

There was a wide range of phenotypic variation among the F_2:3_ populations in all the traits measured and in both environments: MDA, EC; GR; FW; SL; LFW; SLW; DLW; RWC and CHL Figure 1. All traits exhibited a normal segregation pattern, with equal distribution (Figure S1). In the control, there was no significant difference observed between the parents, except for chlorophyll content (CHL). However, under salt treatment in all environments, whether 110 mM or 150 mM, all traits were significantly reduced in the salt-susceptible genotype compared to the resistant parent; MDA, LFW, SLW, GR, and SL showed a significant difference. However, in MDA, higher concentration denoted sensitivity; its concentration in a resistant accession was significantly lower than the sensitive accession (Figure 1).

### 2.4. ANOVA and Heritability Analysis of Salt Tolerance Traits for the Two Parents and the Progeny

All the data collected were analyzed for ANOVA using mixed-model analysis. The ANOVA results revealed significant differences between the genotypes, environment, and their interactions for all the traits (Table 1).

The measured trait’s heritability percentage was much higher on the morphological traits compared to the physiological traits. The highest percentage of heritability was achieved in fresh weight (FW), at 87.4%, while the lowest level of heritability was noted in relative water content (RWC), at 42.3%.

### 2.5. Correlation Analysis

Correlation analysis was carried out using the mean values of the five seedlings in each replicate for the two salt treatments, 110 mM and 150 mM.

#### 2.5.1. Salt Treatment (110 mM)

**Figure 2:**
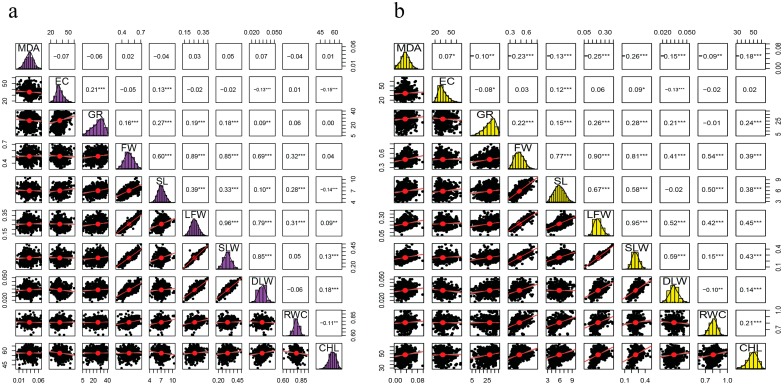
Correlation analysis of the 10 traits in two salt environments:(**a**) 110 mM, (**b**) 150 mM, (*), (**), (***) significant levels of 0.5, 0.01 and 0.001 respectively.

Under 110 mM, a greater percentage of the traits were positively correlated. However, MDA had no significant correlation to any of the measured traits. Negative correlation was noted on EC/DLW, EC/CHL, SL/CHL, and RWC/CHL, while no significant correlation was observed between EC/FW, EC/LFW, EC/SLW, EC/RWC, GR/RWC, GR/CHL, FW/CHL, SLW/RWC, and DLW/RWC (Figure 2a).

#### 2.5.2. Salt Treatment (150 mM)

In an increased salt concentration of 150 mM, MDA was negatively correlated to all traits except for EC, but EC was negatively correlated to GR and DLW. Moreover, GR, FW, SL, LFW, SLW, DLW, RWC, and CHL were positively correlated. However, no correlation was noted between RWC/GR, EC with FW, LFW, RWC and CHL; DLW/SL (Figure 2b).

### 2.6. Construction of the Linkage Maps

**Figure 3:**
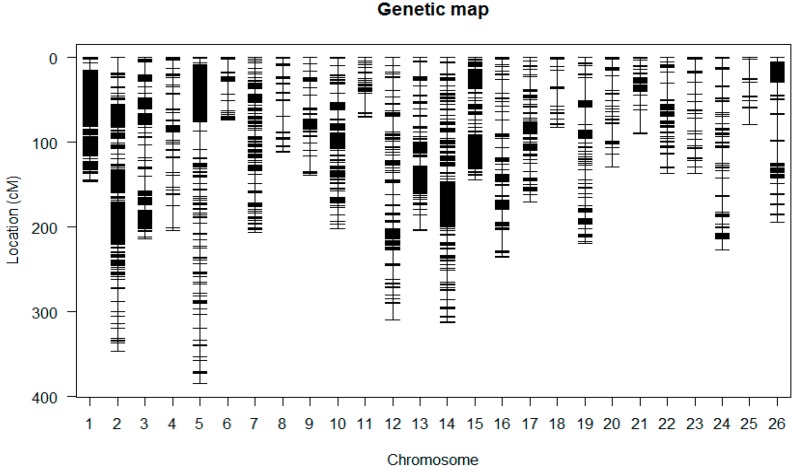
Genetic map constructed using the F_2:3_ population derived from the parental lines.

A total of 6405 markers and phenotypic data of the F_2:3_, an intra-specific cross of two upland cottons, were utilized for generating the intra-specific linkage map. The genotyping data of 5178 GBS markers were used for mapping after removing the distorted and the unlinked markers in Join Map 4.0 (Table S1). Linkage maps of the 5178 GBS markers generated 26 linkage groups (Figure 3 and Figure S2 and Table 2). The 26 LGs were designated as A01 to A13 for the A_t_ sub-genome and D01 to D13 for the D_t_ sub-genome. The total map distance was 4768.098 cM, higher than the most current linkage map with a map distance of 4450 cM of cotton genome [30]. The map developed in this study is the most highly saturated intra-specific map of upland cotton ever developed. The average distance between adjacent markers was 0.92 cM. The A_t_ sub-genome spanned 2611.43 cM and consisted of 3313 markers with 13 linkage groups. The average distance in A_t_ sub-genome was 0.79 cM, with a maximum gap of 26.598 cM between adjacent markers. In the D_t_ sub-genome, 13 linkage groups were assigned, comprised of 1865 markers spanning 2156.67 cM, with an average of 1.156 cM. The maximum gap between adjacent loci was 30.082 cM (Table 2). Chromosomes; A02, D02, A01, A05, A03, D01 and A10 had more markers compared to other chromosomes such as D06 and D13. For instance, Chr A02 had 705 loci with map distance of346.314 cM with average distance of 0.49 cM. Chr D06 was the smallest with only 16markers, and a total length of 79.084 cM. 

### 2.7. Clustered QTLs Region

**Figure 4:**
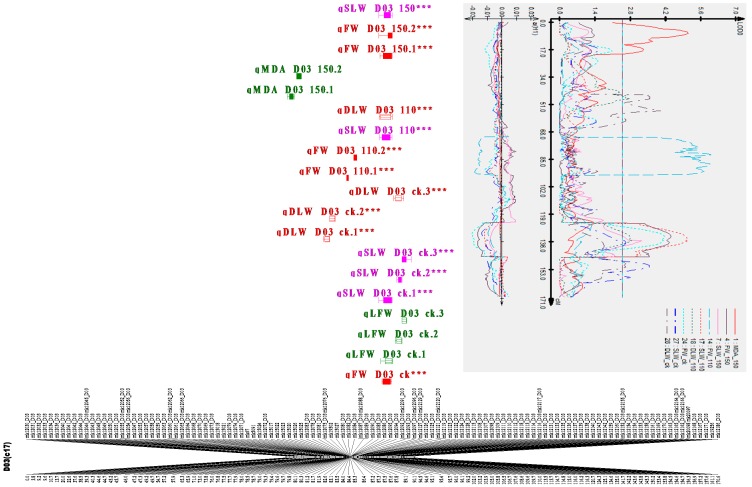
Clustered QTLs identified in D03 (c17) for salt tolerance. Bars and lines on the right-hand side of the linkage groups show the QTL likelihood intervals. Map distances in centimorgan (cM) are indicated at the left-hand side of the linkage groups. Asterisk (***): means the QTL is consistent.

QTL clusters are regions of the genome in which large quantities of QTLs are co-localized [31]. A total of eight clusters for six traits were detected. The highest number of consistent QTLs mapped was three and all were identified in the marker interval of mk12058_D03-mk12186_D03on D03 (c17), as in Figure 4. This region was designated as Cluster 5, from 3,459,294 bp to 42,120,184 bp. Cluster 5 harbored QTLs for DLW, FW, and SLW with the following proportions: 4, 5, and 5, respectively, which explained the phenotypic variance ranging from 2.72% to 9.87% (Table 3 and Table S2). The lowest number of major QTLs was identified in Clusters 1, 7, and 8, which harbored QTLs for EC, FW, and MDA with the following proportions: 5, 3, and 2, respectively. Furthermore, we focused our analysis on the 14 stable QTLs across multiple environments based on their broad-sense heritability and phenotypic variation explained by a single QTL. These QTLs were mapped in eight chromosomes; A02, A06, A12, D01, D03, D06, D08, and D13, with five, six, five, eight, 14, four, three, and two QTLs, respectively (see Table 3 and Figure S2).

A total of 66 QTLs were identified for 10 traits, but only 14 QTLs were consistent in at least two environments (Table S2 and Figure S2). The distribution of the QTLs within the identified chromosomes is illustrated in Table 3. Of the 14 consistent detected QTLs, five were located on the A_t_ sub-genome while the remaining nine were located on the D_t_ sub-genome (Table 3). As to the contributions of the parents toward the QTLs, 10 QTLs were contributed by the sensitive parent (NH), while only four QTLs were contributed by the resistant parent (CCRI35). Moreover, only eight chromosomes out of 26 were found to harbor consistent QTLs for six traits (EC, FW, SLW, SL, DLW, and MDA) related to salt tolerance. Four types of gene actions were revealed by the genetic effects, of which four genes exhibited dominant effects (D), 13 partial dominance (PD), 28 overdominance (OD), while only two had an additive effect (A). OD was observed for most of the traits in response to salt tolerance, as shown in Table 3.

### 2.8. Identification of Candidate Genes

**Figure 5:**
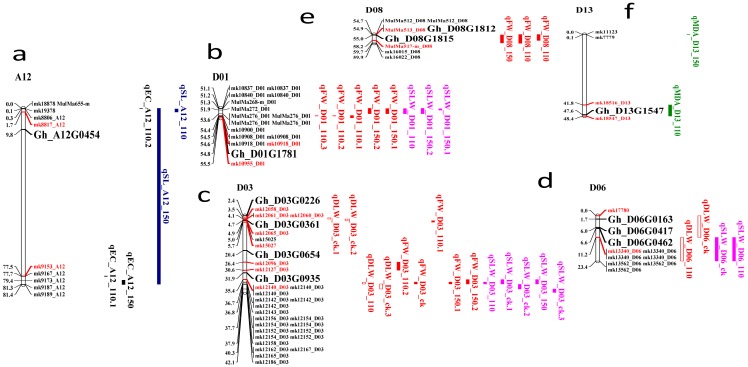
Candidate genes identified by syntenic analysis between two markers. Red loci represent the flanking markers and black represent the key genes. Unit distances in Mb. Chromosomes are as follows: (**a**) A12; (**b**) D01; (**c**) D03; (**d**) D06; (**e**) D08; (**f**) D13.

**Figure 6:**
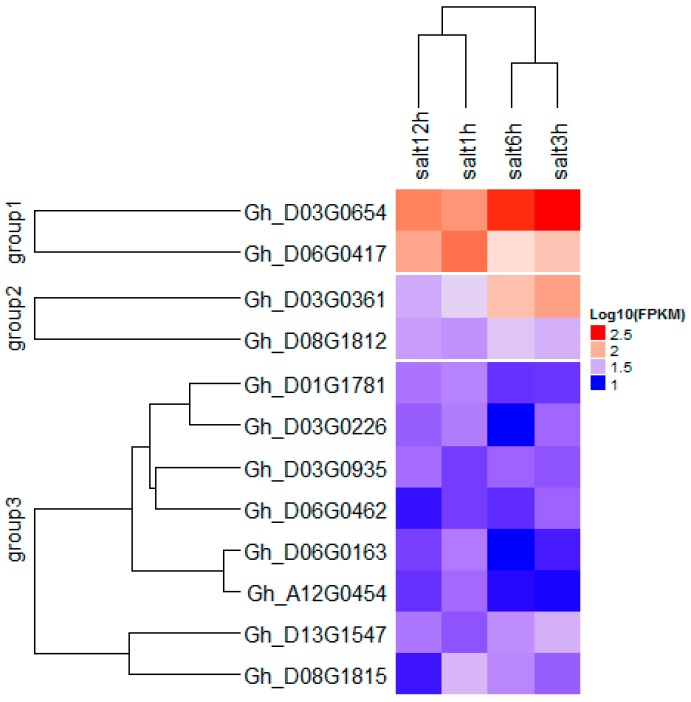
Heat map of identified putative candidate genes in various cotton leaf salt treatment.

The putative candidate genes identification was done based on the QTLs consistency. The overall number of consistent QTLs was grouped into eight clusters with varying marker flanking intervals: Cluster 1 (mk19866 to mk1778_A02); Cluster 2 (mk4886_A06 to mk5000_A06); Cluster 3 (mk18878 to mk9189_A12); Cluster 4 (mk10837_D01 to mk10918_D01); Cluster 5 (mk12058_D03 to mk12186_D03); Cluster 6 (mk17780 to mk13562_D06); Cluster 7 (MulMa512_D08 to mk16015_D08), and finally Cluster 8, which had a marker interval from mk11123 to mk18547_D13. Clusters 1 to 8 were located on chromosomes A02, A06, A12, D01, D03, D06, D08, and D13, respectively. The highest number of stable QTLs was detected in Cluster 5 (D03), with three QTLs linked to DLW, FW, and SLW (see Table S3 and Figure 5 and Figure 6).

In all the clusters, 5596 genes were mined, of which 176 were found to be related to biotic and abiotic stress. The stress-related genes were found to belong to the NAC, WRKY, HAD, C2H2, and RING/U-box superfamily proteins (Table S3). In order to identify the most robust candidate genes for salt tolerance, all 176 genes were analyzed using “TM-1” RNA-seq expression profiles data at different time points of salt treatment 1, 3, 6, and 12 hin leaves part area (http://mascotton.njau.edu.cn). Out of 176 genes, 12 genes were highly expressed in salt treatment (see Table S3 and Figure 5 and Figure 6). Of the 14 major QTLs identified, 12 genes were predicted to be the putative candidate genes associated with salt-tolerant traits.

**Figure 7:**
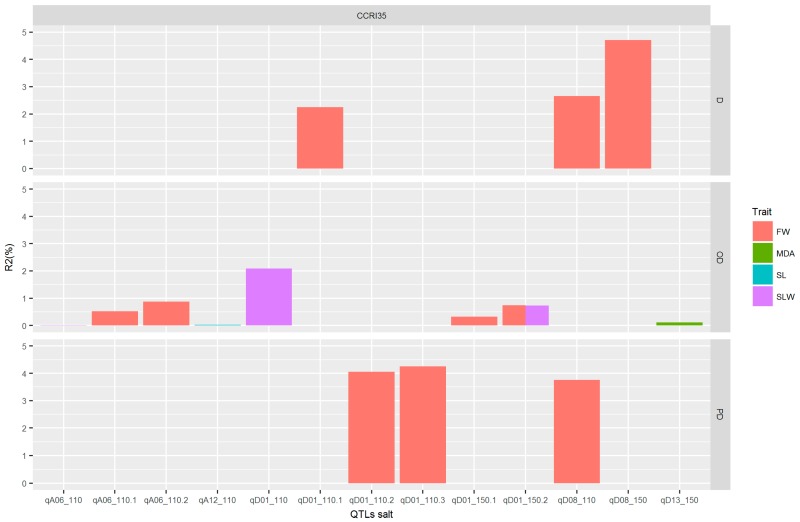
QTLs for salt tolerance. The *x*-axis indicates the QTL for these different traits. The *y*-axis indicates the phenotypic variation explained by the QTL for each trait; FW: Fresh Weight (g); MDA: Malondialdehyde (µM/g.FW); SL: Stem Length (cm), SLW: Saturated Leaves Weight (g), D: dominance effect, OD: overdominance effect and PD: partial dominance effect.

In order to understand which QTL is preferable out of the 14 detected, we based our research on the additive effect and gene action (GA). Therefore, the parental contribution was determined by the use of additive effect. For positive and negative additive effects, alleles came from CCRI35 (the tolerant parent) and NH (the sensitive parent), respectively. GA was obtained from the ratio dominant effect by additive effect, |d/a| (see Table 3). To get good QTLs for salt tolerance, we focused our analysis on the QTLs with positive additive effect, coming from the tolerant parent, CCRI35. Therefore, seven QTLs were involved with the four following traits: FW, MDA, SL, and SLW (Figure 7). The genes’ actions were partial dominance: PD (with FW trait), dominance: D (with FW trait), and overdominance: OD (with MDA, SLW, FW, and SL traits). The highest number of QTLs had an OD effect. This result suggests that these QTLs can be used for further studies in salt tolerance. Moreover, the 12 genes identified were classified into three groups, with two, two, and eight genes in Group1, Group2, and Group3, respectively. The best expression pattern was noted in Group1 (see Figure 6 and Table S3).

### 2.9. Collinearity Analysis

**Figure 8:**
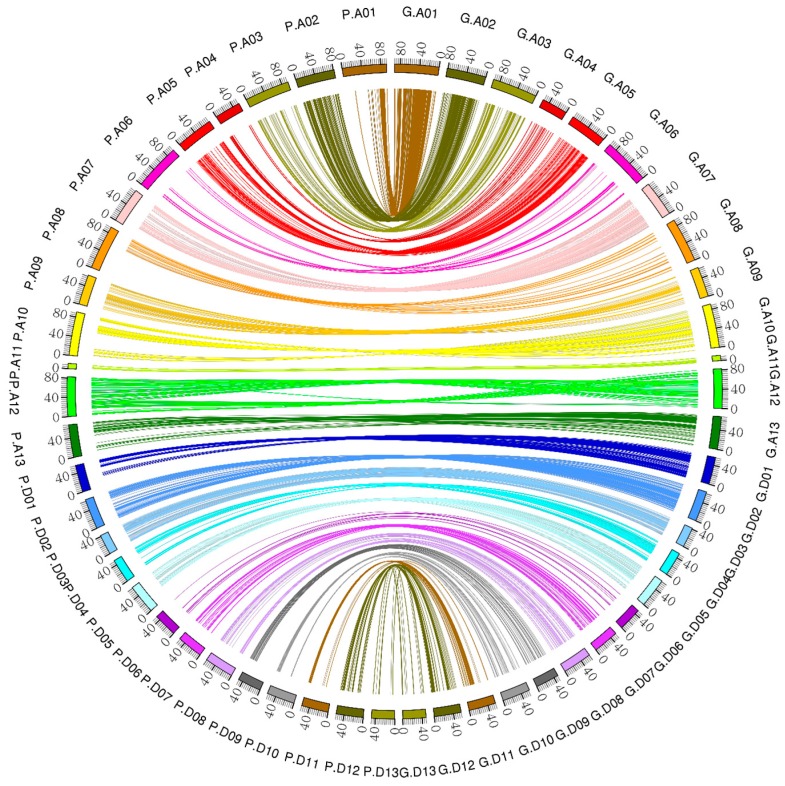
Collinearity between the genetic map and the physical map.

The syntenic blocks were obtained by comparing the 5178 marker position in the genetic map and physical map using AD cotton genome. The results showed that most of the markers had good collinearity (Figure 8). However, some chromosomes, such as A10 and A12, showed poor syntenic blocks. This could be attributed to the mutation effect.

## 3. Discussion

This study was carried out in a controlled environment and the F_2:3_ population and their parents were exposed to salt at the seedling stage since much evidence points to the fact that salt tolerance is a stage-specific phenomenon and seedlings are the most vulnerable to salinity stress [32]. The main aim of this research was to determine the response of cotton seedlings to salt stress when grown in a salt environment. The seedlings were highly assimilated to the natural environment by using soil as the rooting media. Soil substrate type should be considered when characterizing plant growth and physiological responses to salinity [33].

The plant materials used in developing the mapping population had varying salt stress tolerance. Significant differences between the two parents for all the measured traits were observed, both under stress and control conditions. Under stress, all the traits exhibited a significant reduction in LFW, SLW, GR, SL, and MDA. In the sensitive cultivar (NH), MDA concentration was higher than in the tolerant cultivar (CCRI35). MDA is a biochemical component of the cell; its release is triggered by exposure to stress. The lower MDA level in the tolerant cultivar is an indication of the ability of the plant to tolerate salt stress; this result is consistent with previous publications [34,35]. Moreover, the low concentration of MDA in the tolerant cultivar can be attributed to either an internal mechanism to convert the released MDA into other non-toxic compounds or minimizing the release of MDA to a minimal threshold with no major effect on the plant cell. Previous reports have shown that MDA in salt-sensitive plants is more pronounced than in salt-tolerant ones [36,37]. Furthermore, similar results were found by [38], stipulating that with regard to MDA content reduction in tolerant cultivars, it has been shown that low MDA content inhibits membrane damage by ROS and hence confers tolerance [39,40].

Under controlled conditions, in all the measured traits, no significant statistical difference was noted between the tolerant parent (CCRI35) and the sensitive parent (NH), except for CHL concentration. The sensitive cultivar produced more chlorophyll than the tolerant one. The increased level of chlorophyll content in the sensitive cultivar can be explained by the ability of the plant to maximize its photosynthetic apparatus, preparing for any sudden alteration that may occur within its environment, thus enhancing its survival. This result was consistent with the findings of [38], which reported that the chlorophyll (a), chlorophyll (b), and total chlorophyll contents were significantly lower in tolerant cultivars in comparison to sensitive cultivars. Moreover, plants under salt stress conditions are often observed to have reduced chlorophyll content [40,41].

Under stress, five traits showed significant difference (SL, GR, SLW, MDA, and LFW), while the rest showed no significant difference. However, as for chlorophyll concentration (CHL), in all the genotypes, the tolerant cultivar showed relatively higher concentrations in chlorophyll but statistically, no significant difference. It has been reported that salt-tolerant plants exhibit increased or unchanged chlorophyll content under halophytic conditions, whereas chlorophyll concentrations decrease in salt-sensitive species, indicating that this parameter can be considered a biochemical marker of salt tolerance in plants [42,43]. The higher number of chloroplasts per leaf area unit may be the main reason for the increased chlorophyll concentration in tolerant plants under salt stress. However, in the sensitive cultivar, salt stress may lead to stomatal closure, which in turn reduces the availability of carbon (IV) oxide in the leaves and therefore inhibits carbon fixation, exposing chloroplasts to excessive excitation energy, which in turn could increase the generation of reactive oxygen species (for example, MDA or other toxic elements) and induce oxidative stress [44]. High accumulation of sodium in plant tissues has been reported as one of the factors contributing to the reduction of photosynthetic pigments and rate of photosynthesis [45,46]. Chlorophyll is an important factor in plant photosynthesis; long exposure to salt stress may lead to the impairing of the photosynthetic apparatus of the plant, and in turn lead to a reduction in the plant’s performance in terms of yield and the quality of the salt-sensitive cultivars.

Most of the traits were positively correlated. MDA was negatively correlated to all traits in 150 mM of salt treatment but no significant correlation was noted in 110 mM of salt treatment. Plant growth and development are impaired with an increase in salt concentration [47]. Previous studies have shown that 150 mM NaCl results in significant growth reduction between the salt-tolerant and less salt-tolerant cotton cultivars [48,49]. A negative correlation was observed between EC and the following traits; GR, DLW, and DLW with RWC at 150 mM salt concentration. The results obtained are in agreement with previous findings in *Brassica napus* under salt stress: EC was significantly negatively correlated with RL, RDW, and SFW [50].

The extent of transmission of traits from the parents to the offspring is determined by levels of heritability and hence traits with higher heritability percentage are easier to manipulate [51]. The heritability was high for FW (87.4), SL (83.1), LFW (87.3), SLW (86.8), and DLW (82.6), moderate for MDA (73.4), EC (57.5), and GR (68.4), and lowest in CHL (51) and RWC (42.3). These results were in agreement with previous reports that indicated that heritability estimates in cotton are moderate to high for SH, RL, RDW, SFW, RFW, and SDW under salt conditions [52]. Selection, done based on low heritability, may be biased towards environmental influence on the genetic make-up of the plant and thus is not an effective way to improve plants [53]. In this study, the number of QTLs identified for high heritability traits was consistent, except for LFW.

Transgression was also a phenomenon noted in plant species. Ten QTLs were contributed by the sensitive parent (NH), while only four were contributed by the resistant parent (CCRI35). The positive and negative signs of the additive effect at the different loci showed the parental lines’ contribution and confirmed the transgressive segregation pattern observed at the phenotypic level. Transgressive segregation for morphological and agronomical traits always follows the Mendelian pattern [54]. This observation was also reported by [55,56] in RILs population of *Arabidopsis thaliana* cultivated in a nitrogen environment.

In this study, four types of gene actions (GA) were revealed by the genetic effects: four genes exhibited dominant effects (D), 13 partial dominance (PD), 28 overdominance (OD) and two had an additive effect (A). Overdominance (OD) was observed for most of the traits in response to salt tolerance. This result was in disagreement with Oluoch et al. (2016) [25], who stated that partial dominance (PD) was observed for most traits in response to salt tolerance. In Oluoch et al. (2006), the marker and map size were relatively smaller than in our map; their final linkage map consisted of 1295 markers that amplified 1342 loci [25], and this could hamper the effective determination of the gene action identification. In this study, we developed a high-density linkage map with ≤1 cM of marker intervals, which enabled us to identify QTLs with more accuracy and higher resolution than earlier reports.

A total of 14 consistent QTLs for six traits were detected, which explained the phenotypic variance ranging from 2.72% to 9.87%. Five QTLs were located in the A_t_ sub-genome, while the remaining nine were located in the D_t_ sub-genome. This finding was consistent with earlier reports [25,57] that suggested that the D_t_ sub-genome harbored genes or QTLs for stress biotic, abiotic, and fiber quality. Oluoch et al. (2016) stipulated that, of the 11 significant QTLs detected, only one (qSdw-Chr9-1) on chromosome 9 was located in the A_t_ sub-genome, while the remaining 10 were located in the D_t_ sub-genome. Meanwhile, approximately 58 QTLs were identified on the A_t_ sub-genome chromosomes, whereas 107 QTLs were localized on the D_t_ sub-genome chromosomes [57]. Saeed et al. (2014) highlighted the contribution of the D_t_ sub-genome of tetraploid cotton to abiotic stress tolerance [14]. QTLs were also present at unique loci with defined underlying markers on different chromosomes, and none were shared by two or more traits. However, some were present in close proximity to each other, such as QTLs on chromosome D03 with the following traits: MDA and FW.

A number of the QTLs found in this study were in agreement with previous findings [14]. For example, Clusters 2 (A06), 6 (D06), and 7 (D08), according to Saeed et al. (2014) [14], were reported to be flanked by markers NAU2679 (A06), BNL3103 (D06), and NAU478 (D08), respectively, all with significant associations with salt treatment.

Of the 12 key genes found in this study, 11 genes were located in the D_t_ sub-genome. Only *Gh_A12G0454* gene on chromosome A12, which belongs to the zinc finger (C2H2 type) family protein, was located in the A_t_ sub-genome. This result could explain the high number of QTLs in the D_t_ sub-genome compared to the A_t_ sub-genome in upland cotton. This finding is consistent with reports by Oluoch et al. (2016) and Jamshed et al. (2016) [25,57]. Moreover, the 12 key genes were localized in six families: RING/U-box superfamily protein, NAC, WRKY, Haloacid dehalogenase-like hydrolase (HAD) superfamily protein, zinc finger (C2H2 type) family protein, and MYB. *Gh_D03G0935*, *Gh_D06G0163*, and *Gh_D06G0462* genes belonged to RING/U-box superfamily protein and were located on Clusters 5 and 6, respectively. The U-box genes in Arabidopsis such as *AtPUB23* have been reported to show strong upregulation in the roots under salt and drought conditions [58]. The highest expression was observed with *Gh_D03G0654* and *Gh_D06G0417* genes in D03 and D06, respectively (Figure 6 and Table S3). These two genes belonged to Group1 and could be helpful in further salt-tolerant gene studies. Furthermore, C_2_H_2_ (94 genes), MYB (225 genes), NAC (170 genes), and WRKY (222 genes) unigenes were mostly upregulated under salt stress. MYB, NAC, and WRKY were also highly enriched at 4 h and 24 h of salt stress [15].

## 4. Materials and Methods

### 4.1. Greenhouse Experiment

Seeds of two upland cotton (*G. hirsutum*) lines, CCRI35 (salt-tolerant) and Nan Dan Ba Di Da Hua or NH (salt-sensitive) were used. The salt-tolerant cultivar was used as the female plant while the salt-sensitive one was the male plant. All plant materials were obtained from the National Mid-Term Gene Bank of the Institute of Cotton Research, Chinese Academy of Agricultural Sciences [59]. The seeds were then sterilized in 70% ethanol for 15 s, and put in 4% sodium hypochlorite for 15 min. The seeds were later submerged in sterile water for 12 h to rinse off the treatment chemicals. The seeds were sown in sterile silica sand with 0, 110, and 150 mM salt concentrations in planting boxes measuring 60 cm by 35 cm and depths of 12 cm in a greenhouse, where the conditions were optimized to 28/22 °C day/night, 60–80% relative humidity, and a 14-h photoperiod under 450 µmol·m^−2^·s light intensity. The experiment was done by laying out a complete randomized block design (CRD) with three replicates, with 10 and four seeds sown in 60 cm long and 35 cm wide rows, respectively. Two experimental periods were chosen, the first one from April to June 2015, and the second one from April to June 2016, corresponding to the cotton cultivation seasons in the Yellow River region of China with different salt concentrations of 110 mM or 150 mM. In both experiments, the plant population size was 277, with three replications. The two salt concentrations, 110 mM and 150 mM, were chosen based on the proportion of Hoagland [60]. Seven days after sowing (D.A.S), five seedlings with uniform growth for each treatment were sampled for all traits. Seed germination (GR) was determined by counting the seedlings in each replication. Five fresh seedlings were weighed for fresh weight (FW) in each replicate; leaf fresh weight (LFW) and stem length (SL) were measured immediately after collection. The sampled leaves were submerged in distilled water overnight and their saturated leaves were weighed (SLW). The leaf samples were then oven dried at 60 °C for 48 h; after attaining constant mass, all were weighed to obtain the dry leaf weight (DLW). Relative water content (RWC) was calculated using the formula described by [61]. The electric conductivity (EC) of the sample leaves was determined by using a sliced leaf portion of 0.5 g dipped in 100 mL of ddH_2_O; initial measurements were taken at the start, while the final measurements were taken after 48 h, as explained by [62]. For chlorophyll content (CHL) estimation, we used a chlorophyll meter (SPAD 502 m, Minolta, Osaka, Japan), and the average of five measurements on the same leaf was taken.

### 4.2. Sample Collection, Library Preparation, Sequencing, and SNP Genotyping

#### 4.2.1. DNA Quantification and Qualification

The DNA of the entire population of 277 individuals of the F_2:3_ generation, together with 10 samples for each parent, was extracted by the CTAB method [63]. Fresh and young leaves were collected and immediately frozen in liquid nitrogen. Each sample was then crushed in liquid nitrogen into a fine powder, then immediately added to a CTAB solution. For each 100 mg of homogenized tissue, we used 500 µL of CTAB extraction buffer. The contents were mixed and thoroughly vortexed. The homogenized mixture was then incubated in a 60 °C water bath for 30 min. Following the incubation period, we centrifuged the homogenate for 5 min at 12,000× *g*. After centrifuging, the supernatant was transferred to a new tube. Then 5 µL of RNase solution was added to remove RNA and the result was incubated at 32 °C for 20 min. An equal volume of chloroform/isoamyl alcohol (24:1) was added and the sample was vortexed for 5 sand centrifuged for 1 min at 12,000× *g* to separate the phases. We transferred the aqueous upper phase to a new tube; the method was then repeated until the upper phase was clear. The upper aqueous phase was then transferred to a new tube. DNA was precipitated by adding 70% volume pre-refrigerated isopropanol and incubating at −20 °C for 15 min. The precipitated DNA samples were then centrifuged at 12,000× *g* for 10 min. The supernatant was then decanted without disturbing the pellet and subsequently washed with 500 µL ice cold 70% ethanol twice, then absolute alcohol. Then DNA pellets were later dissolved in 20 µL TE buffer (10 mM Tris, pH 8, 1 mM EDTA) [64]. DNA degradation and contamination were monitored on 1% agarose gels. DNA purity was checked using the Nano Photometer^®^ spectrophotometer (IMPLEN, Westlake Village, CA, USA). The ratio of absorbance at 260 nm and 280 nm was used to assess the purity of DNA. The DNA samples with a ratio of ~1.8 were selected as pure [65]. The DNA concentration was measured using Qubit^®^ DNA Assay Kit in Qubit^®^ 2.0 Fluorimeter (Life Technologies, Carlsbad, CA, USA). The Qubit^®^ dsDNA HS (High Sensitivity) Assay Kits make DNA quantitation easy and accurate. The kits include concentrated assay reagent, dilution buffer, and pre-diluted DNA standards. The reagents were diluted by the buffer solution, then we added 1–20 μL of each DNA sample. The concentrations were read using the Qubit^®^ Fluorometer; only DNA samples with a concentration range of 10 pg/µL to 100 ng/µL were used (https://tools.thermofisher.com/content/sfs/manuals/Qubit_dsDNA_HS_Assay_UG.pdf).

#### 4.2.2. GBS Library Preparation, Sequencing, and SNP Genotyping

Genotyping-by-sequencing (GBS) is an efficient method of large-scale genotyping, which is based on reduced-representation libraries (RRL) and high-throughput sequencing. First, we performed a GBS pre-design experiment to confirm the effectiveness of the GBS protocol and the quality of the output data. The enzymes and sizes of restriction fragments were evaluated using training data. Three criteria were considered: (i) The number of tags must be suitable for the specific needs of the research project; (ii) the enzymatic tags must be evenly distributed through the sequences to be examined; (iii) repeated tags must be avoided. These considerations improved the efficiency of GBS. To maintain the sequence depth uniformity of different fragments, a narrow length range was selected (about 50 bp).

Next, we constructed the GBS library in accordance with the pre-designed scheme. For the F_2:3_ populations, genomic DNA was incubated at 37 °C with MseI (New England Biolabs, NEB, Ipswich, MA, USA), T_4_ DNA ligase [66], ATP [66], and MseI Y adapter N containing barcode. Restriction-ligation reactions were heat-inactivated at 65 °C and then digested for additional restriction enzyme NlaIII at 37 °C. The restriction ligation samples were purified with Agencourt AMPure XP (Beckman, Brea, CA, USA). Then a PCR reaction was performed using purified samples, Phusion Master Mix [66] universal primer, and index primer to add index, complete i5 and i7 sequence. The PCR productions were purified using Agencourt AMPure XP (Beckman) and pooled, then run out on a 2% agarose gel. Fragments with 375–400 bp (with indexes and adaptors) in size were isolated using a Gel Extraction Kit (Qiagen, Hilden, Germany). These fragment products were then purified using Agencourt AMPure XP (Beckman), which was diluted for sequencing.

Genotyping-by-sequencing (GBS) was carried out as outlined by Elshire et al. (2011) [67], integrating three 96-well plates across 288 barcodes for library preparation and sequencing. For SNP calling, the raw sequence data for the 277 F2 progeny plus the F1 progenitor were processed through the TASSEL 3.0 GBS pipeline [68] using *Gossypium_hirsutum_v1.1.fa* as the reference genome [69], obtained from Nanjing Agriculture University’s Cotton Research Institute (http://mascotton.njau.edu.cn/info/1054/1118.htm) for alignment and the Burrows–Wheeler Aligner (BWA) mem [70] with default parameters. The output consisted of variant call format (VCF) file version 4.1 [71], including SNPs present in at least 40% of the progeny and with a minor allele frequency (MAF) 0.1. Subsequently, the VCF was filtered using vcf tools version1.12a [71] and TASSEL [72] versions 3.0 and 4.0. A total of 93,384 SNPs were identified in 277 F_2:3_ progeny by TASSEL 3.0, then a custom filtering process was applied for alignment. The filtering was based on keeping sites with a minimum read depth of 6% and 75% completeness by site across progeny and by progeny across sites. The results were produced as a TASSEL hapmap file.

Finally, using custom perl script markers heterozygous in the F1 progenitor and witha co-dominant 1:2:1 segregation among the F_2:3_, progenies were identified by a chi-squared (χ^2^) goodness-of-fit test at α ≤ 0.01. These were reformatted to be imported in JoinMap^®^ 4.1 [73] for linkage group determination. A total of 26 linkage groups were formed; each linkage group was matched to its corresponding chromosome using BLASTN for the marker sequence (https://blast.ncbi.nlm.nih.gov/Blast.cgi).

### 4.3. Construction of the Linkage Maps and Data Analysis

ANOVA was performed using the greenhouse experiment data of the two seasons, 2015 and 2016. A mixed procedure was used; the genotypes and the environments were fixed as factors in order to detect the heritability. A post hoc test (Turkey’s) was used to compare means. The broad-sense heritability (H) was calculated using the formula described by [31]:

H = σ^2^G/σ^2^G + (σ^2^e/r)

where σ^2^G is the genotypic variance; σ^2^e: phenotypic variance and r: replication. All the data were analyzed using R software version 3.4.1 [74].

Linkage map analysis was conducted using Join Map 4.0 [73] with a recombination frequency of 0.40 and a LOD score of 2.5 for the F_2:3_ population. The Kosambi mapping function was used to convert the recombination frequencies to map distances. For the two experiments, each data point represented the mean of three replications, with each treatment consisting of 40 plants. Salt-tolerance-related traits EC, GR, SL, FW, LFW, SLW, DLW, RWC, and CHL were used to conduct a QTL analysis. WinQTL Cartographer 2.5 was used to detect QTLs using composite interval mapping by [75]. In the composite interval mapping (CIM) method, model 6, forward–backward regression method with 1 cM walking speed, a probability into and out of the model of 0.01, and window size set at 10 cM were used. A stringent logarithm of odds (LOD) threshold value was estimated by 1000 permutation test for all traits and used to determine the significant QTLs with a significance level of *p* = 0.05. However, QTLs in varying environments with an LOD threshold of at least 2.5 were considered common QTLs based on the explanation by Lander and Kruglyak [76].

QTL nomenclature was done based on previous criteria [77]. The phenotypic variance observed proportions as illustrated by each QTL was estimated by coefficient of determination R^2^ (%) as a percentage. The additive and dominance effects from QTL cartographer results were used to calculate the genetic effects (|d/a|). The results were used to classify the QTL as additive (A) (0–0.20), partially dominant (PD) (0.21–0.80), dominant (D) (0.81–1.20), or over-dominant (OD) >1.20, according to [78]. The graphic presentation of the linkage group and QTLs marked was created by Map Chart 2.2 [79] and the R/qtl package was used to generate the genetic map in R software version 3.4.1 [74].

In this study, only consistent QTLs were used to identify the crucial candidate genes for salinity tolerance. The genes were identified through the available resources [80] (http://mascotton.njau.edu.cn). The physical position of the SNP markers flanking major QTLs for salinity tolerance was used to find the genes located in each cluster. The function of the identified genes was determined through gene annotation. Furthermore, the expression profiles of the candidate genes were analyzed by mapping the genes in the “TM-1” RNA-seq transcriptome data of cotton leaf tissues at different time points of salt treatment (1, 2, 3, 6, and 12 h) (http://mascotton.njau.edu.cn). The putative candidate genes were mapped using VLOOK UP Excel formula and the output FPKM for each gene was expressed to log10 to get the final gene expression. These values were used to generate the heat map by using R script.

### 4.4. Collinearity Analysis

Based on the high-density genetic linkage map and sequences corresponding to markers, collinearity analysis between genetic and physical maps within chromosome was carried out using a BLASTN (https://blast.ncbi.nlm.nih.gov/Blast.cgi) search with E ≤ 1 × 10^5^, identity ≥80%, and matched length ≥200 bp. Next, the best hit for each marker was chosen and all the best hits were illustrated intuitively using online drawing tools (http://circos.ca/).

## 5. Conclusions

We identified 66 QTLs, of which 14 QTLs were consistent in eight chromosomes in at least two environments. A total of 5596 genes were identified; 12 genes showed preferential expression in leaf tissues at different time points (1, 3, 6, and 12 h) of salt treatment in the “TM-1” transcriptome data. The genes found in this research work were in three groups (Figure 6); 11 genes were from the D_t_ sub-genome, and only one (*Gh_A12G0454* in chromosome A12) was identified from the A_t_ sub-genome. Cloning and gene saturation need to be done to verify their specific functional role in cotton plants under salt stress.

## Figures and Tables

**Table 1:** ANOVA and heritability analysis of salt tolerance traits for the two parents and the progeny.

F_2:3_																	
Trait ^a^	Env ^b^	Source	Df	Mean Square	F Value	Pr > F	H_B_ (%)	NH = P2	CCRI35 = P1	P1–P2	Mean	Var	SD	Min	Max	Skew	Kurt
**MDA**	CK	e	2	0.009	199.712	<0.0001	73.4	0.028	0.022	−0.006	0.042	0	0.011	0	0.085	0.052	0.753
	1	g	276	0.001	14.401	<0.0001		0.027	0.015	−0.012	0.037	0	0.011	0.004	0.066	−0.05	0.159
	2	g * e	543	0.0003	6.11	<0.0001		0.014	0.009	−0.005	0.036	0	0.016	0	0.098	0.198	0.081
**EC**	CK	e	2	29,102.2	978.452	<0.0001	57.5	68.505	64.085	−4.42	45.469	109.207	10.45	22.754	92.32	0.72	0.762
	1	g	276	246.072	8.273	<0.0001		37.882	41.406	3.524	35.448	41.403	6.435	18.824	59.902	0.63	0.645
	2	g * e	543	125.338	4.214	<0.0001		39.695	41.617	1.922	34.601	75.858	8.71	16.713	67.505	0.966	0.883
**GR**	CK	e	2	8313.01	427.302	<0.0001	68.4	31	33.667	2.667	32.922	33.583	5.795	7	40	−1.17	1.364
	1	g	276	191.801	9.859	<0.0001		22.667	26.5	3.833	26.677	61.173	7.821	6	40	−0.533	−0.535
	2	g * e	543	65.441	3.364	<0.0001		26	30	4	28.355	54.829	7.405	4	40	−0.774	0.058
**FW**	CK	e	2	8.543	7435	<0.0001	87.4	0.615	0.646	0.031	0.668	0.007	0.086	0.412	0.894	−0.055	−0.394
	1	g	276	0.033	28.453	<0.0001		0.501	0.55	0.049	0.513	0.005	0.071	0.318	0.7	0.053	−0.343
	2	g * e	543	0.009	7.621	<0.0001		0.419	0.445	0.026	0.472	0.007	0.085	0.284	0.79	0.444	−0.029
**SL**	CK	e	2	971.272	6639.29	<0.0001	83.1	9.14	9.267	0.127	8.082	0.512	0.716	5.46	10.54	−0.269	0.407
	1	g	276	3.988	27.261	<0.0001		7.38	8.187	0.807	7.06	0.882	0.939	4.12	10.44	0.107	0.194
	2	g * e	543	1.833	12.526	<0.0001		5.533	6.32	0.787	5.852	1.575	1.255	2.9	9.58	0.172	−0.388
**LFW**	CK	e	2	1.103	3127.67	<0.0001	87.3	0.263	0.305	0.042	0.292	0.002	0.042	0.168	0.416	−0.019	−0.21
	1	g	276	0.01	27.156	<0.0001		0.242	0.273	0.031	0.258	0.002	0.042	0.138	0.396	0.205	0.064
	2	g * e	543	0.002	6.484	<0.0001		0.188	0.216	0.028	0.217	0.002	0.046	0.062	0.39	0.449	0.385
**SLW**	CK	e	2	0.778	1641.26	<0.0001	86.8	0.292	0.343	0.051	0.311	0.002	0.044	0.178	0.432	−0.035	−0.152
	1	g	276	0.012	25.249	<0.0001		0.297	0.329	0.032	0.319	0.002	0.049	0.18	0.494	0.147	0.037
	2	g * e	543	0.003	5.455	<0.0001		0.255	0.285	0.03	0.26	0.003	0.05	0.076	0.478	0.398	0.657
**DLW**	CK	e	2	0.001	86.178	<0.0001	82.6	0.025	0.031	0.006	0.032	0	0.005	0.016	0.046	−0.015	−0.053
	1	g	276	0.0001	17.117	<0.0001		0.032	0.033	0.001	0.034	0	0.005	0.018	0.05	0.044	0.113
	2	g * e	543	2.18 × 10^−5^	2.826	<0.0001		0.037	0.032	−0.005	0.034	0	0.005	0.018	0.052	0.145	0.02
**RWC**	CK	e	2	5.091	7581.42	<0.0001	42.3	0.889	0.878	−0.011	0.934	0.002	0.042	0.524	0.994	−3.565	0.22
	1	g	276	0.006	8.758	<0.0001		0.792	0.809	0.017	0.786	0.001	0.038	0.608	0.941	−0.138	1.348
	2	g * e	543	0.004	6.559	<0.0001		0.691	0.727	0.036	0.807	0.003	0.057	0.615	0.987	−0.141	0.969
**CHL**	CK	e	2	15,495.4	2024.51	<0.0001	51	59.633	52.86	−6.773	54.091	15.969	3.996	39.58	65.82	−0.173	0.345
	1	g	276	67.297	8.793	<0.0001		51.073	51.907	0.834	57.583	14.45	3.801	41.44	66.78	−0.486	0.467
	2	g * e	543	43.419	5.673	<0.0001		48.413	50.94	2.527	48.721	38.069	6.17	29.74	63.46	−0.53	0.126

CK = 0 mM; 1 = 110 mM; 2 = 150 mM; MDA: Malondialdehyde; EC: Electric Conductivity; GR: Germination Rate; FW: Fresh Weight; SL: Stem Length; LFW: Leaves Fresh Weight; SLW: Saturated Leaves Weight; DLW: Dry Leaves Weight; RWC: Related Water Content and CHL: Chlorophyll. For the traits units see Figure 1, Var: Variance, SD: Standard deviation, Min: Minimum, Max: Maximum, Skew: Skewness, Kurt: Kurtosis, ^a^: traits, ^b^: treatments, e: environment, g: genotype, and (*) means interaction. For traits units see to Figure 1.

**Table 2:** Genomic distributions of SNP markers in the AD genome.

Group	Marker_Number	Map_Length (cM)	Av_Distance (cM)	Max_Gap (cM)	<5 cM	5–10 cM	10–20 cM	>20 cM	Ratio
**A01(c1)**	448	146.704	0.33	8.505	445	2	0	0	1
**A02(c2)**	705	346.314	0.49	17.848	687	12	5	0	0.98
**A03(c3)**	323	213.937	0.66	17.145	309	10	3	0	0.96
**A04(c4)**	106	203.891	1.92	26.598	91	8	5	1	0.87
**A05(c5)**	378	385.092	1.02	21.198	354	11	11	1	0.94
**A06(c6)**	58	73.063	1.26	15.032	53	1	3	0	0.93
**A07(c7)**	279	205.892	0.74	11.622	272	4	2	0	0.98
**A08(c8)**	69	112.137	1.63	18.894	58	7	3	0	0.85
**A09(c9)**	98	138.501	1.41	19.234	86	9	2	0	0.89
**A10(c10)**	292	202.134	0.69	10.551	280	7	4	0	0.96
**A11(c11)**	51	70.548	1.38	23.241	47	2	0	1	0.94
**A12(c12)**	244	309.608	1.27	19.593	224	12	7	0	0.92
**A13(c13)**	262	203.61	0.78	17.425	249	7	5	0	0.95
**At sub-genome**	**3313**	**2611.43**	**0.79**	**26.598**	**3155**	**92**	**50**	**3**	**0.94**
**D01(c15)**	319	144.092	0.45	6.351	316	2	0	0	0.99
**D02(c14)**	454	313.268	0.69	14.541	438	12	3	0	0.97
**D03(c17)**	133	170.555	1.28	14.993	124	7	1	0	0.94
**D04(c22)**	114	136.228	1.19	20.275	107	3	2	1	0.95
**D05(c19)**	153	218.788	1.43	27.062	141	7	2	2	0.93
**D06(c25)**	16	79.084	4.94	22.389	11	1	2	1	0.73
**D07(c16)**	169	235.366	1.39	26.041	154	7	6	1	0.92
**D08(c24)**	118	226.688	1.92	20.878	103	6	7	1	0.88
**D09(c23)**	40	136.744	3.42	14.48	28	5	6	0	0.72
**D10(c20)**	80	129.051	1.61	20.539	71	5	2	1	0.9
**D11(c21)**	98	89.782	0.92	27.564	93	2	1	1	0.96
**D12(c26)**	143	194.735	1.36	30.082	133	2	5	2	0.94
**D13(c18)**	28	82.286	2.94	20.917	22	3	1	1	0.81
**Dt sub-genome**	**1865**	**2156.67**	**1.156**	**30.082**	**1741**	**62**	**38**	**11**	**0.9**
**Total (At + Dt)**	**5178**	**4768.1**	**0.92**	**30.082**	**4896**	**154**	**88**	**14**	**0.92**

Ratio: number of markers less than (<) 5 cM divided by total number of markers within chromosome.

**Table 3:** Consistent and clustered QTLs of salt tolerance traits identified in three environments by CIM.

Cluster	Trait	QTLs	Chr	Start Marker	End Marker	Start Marker (bp)	End Marker (bp)	Length of CI Markers (Mb)	Position (cM)	LOD	Ae	De	|d/a|	R^2^ (%)	GA	DPE
**1**	EC	qEC_A02_ck	A02	mk1046_A02	mk1107_A02	4,445,557	7,584,839	3.14		2.64	−1.3522	4.3713	3.23	3.65	OD	NH
		qEC_A02_150	A02	MulMa26-m_A02	mk1778_A02	76,134,216	81,766,125	5.63	20.81	2.78	−1.3853	−2.2243	1.61	1.73	OD	NH
		qEC_A02_110.2	A02	mk19866	mk14259	1876	2247	0.0004	197.41	2.71	−7.1631	−2.5385	0.35	1.14	PD	NH
		qEC_A02_110.3	A02	mk1293_A02	mk1512_A02	23,817,680	40,364,786	16.55	220.01	3.1	−9.7207	−2.9306	0.3	0	PD	NH
		qEC_A02_110.1	A02	mk1340_A02	mk1443_A02	25,911,715	35,098,219	9.19	134.51	2.85	−8.6005	−3.3756	0.39	0	PD	NH
**2**	FW	qFW_A06_150.1	A06	mk4886_A06	mk4999_A06	92,310,429	97,063,193	4.75	0.31	3.71	−0.0093	0.0504	5.42	3.39	OD	NH
		qFW_A06_150.2	A06	mk4886_A06	mk4999_A06	92,310,429	97,063,193	4.75	0.81	4.5	−0.0041	0.0325	7.93	3.14	OD	NH
		qFW_A06_110.1	A06	mk4886_A06	mk4999_A06	92,310,429	97,063,193	4.75	1.31	3.32	0.0019	0.0432	22.74	0.52	OD	CCRI35
		qFW_A06_110.2	A06	mk4998_A06	mk5001_A06	97,063,190	97,172,480	0.11	1.31	4.75	0.0004	0.0294	73.5	0.87	OD	CCRI35
	SLW	qSLW_A06_110	A06	mk4886_A06	mk4999_A06	92,310,429	97,063,193	4.75	1.31	3.05	0.0035	0.0277	7.91	0.01	OD	CCRI35
		qSLW_A06_150	A06	mk4999_A06	mk5000_A06	97,063,193	97,137,676	0.07	0.81	4.5	−0.0038	0.0359	9.45	2.76	OD	NH
**3**	EC	qEC_A12_110.1	A12	mk9153_A12	mk9167_A12	77,455,444	77,705,877	0.25	57.51	4.35	−1.4649	2.4503	1.67	7.91	OD	NH
		qEC_A12_150	A12	mk9173_A12	mk9189_A12	79,355,806	81,365,007	2.01	29.51	3.01	−1.9851	−0.6644	0.33	4.47	PD	NH
		qEC_A12_110.2	A12	MulMa655-m	mk19378	23,988	92,678	0.07	75.21	5.28	−1.2555	4.3961	3.5	8.29	OD	NH
	SL	qSL_A12_150	A12	mk18878	mk9187_A12	173	81,262,301	81.26	46.51	2.68	−0.2876	−0.0593	0.21	4.18	PD	NH
		qSL_A12_110	A12	mk8806_A12	mk8817_A12	253,901	1,660,995	1.41	287.81	2.64	0.065	0.456	7.02	0.03	OD	CCRI35
**4**	FW	qFW_D01_110.3	D01	mk10908_D01	mk10918_D01	54,507,760	54,630,017	0.12	55.41	3.95	0.0097	0.0064	0.66	4.25	PD	CCRI35
		qFW_D01_110.2	D01	mk10908_D01	mk10918_D01	54,507,760	54,630,017	0.12	55.41	3.32	0.0157	0.0072	0.46	4.06	PD	CCRI35
		qFW_D01_110.1	D01	mk10900_D01	mk10955_D01	54,444,280	55,458,818	1.01	50.21	2.51	0.013	0.0127	0.98	2.25	D	CCRI35
		qFW_D01_150.2	D01	mk10837_D01	MulMa276_D01	51,102,990	53,566,034	2.46	82.31	2.72	0.0065	0.019	2.92	0.74	OD	CCRI35
		qFW_D01_150.1	D01	mk10840_D01	MulMa276_D01	51,199,333	53,566,034	2.37	83.31	2.69	0.0097	0.0385	3.97	0.32	OD	CCRI35
	SLW	qSLW_D01_110	D01	mk10840_D01	MulMa276_D01	51,199,333	53,566,034	2.37	80.71	3.37	0.0095	0.0193	2.03	2.09	OD	CCRI35
		qSLW_D01_150.2	D01	mk10837_D01	MulMa276_D01	51,102,990	53,566,034	2.46	82.31	2.84	0.0072	0.0218	3.03	0.73	OD	CCRI35
		qSLW_D01_150.1	D01	MulMa268-m_D01	MulMa272_D01	51,320,058	51,944,113	0.62		2.53	−0.01	0.0227	2.27	4.94	OD	NH
**5**	DLW	qDLW_D03_ck.1	D03	mk12058_D03	mk12060_D03	3,459,294	4,072,981	0.61	54.71	4.04	−0.001	0.0016	1.6	6.92	OD	NH
		qDLW_D03_ck.2	D03	mk12061_D03	mk12065_D03	4,073,116	4,904,755	0.83	60.91	2.65	−0.0007	0.0018	2.57	4.29	OD	NH
		qDLW_D03_110	D03	mk12143_D03	mk12152_D03	36,784,173	37,665,167	0.88	135.01	2.88	−0.0011	0.0001	0.09	4.29	A	NH
		qDLW_D03_ck.3	D03	mk12156_D03	mk12167_D03	37,695,156	40,261,578	2.57	149.31	2.66	−0.0006	0.0019	3.17	3.34	OD	NH
	FW	qFW_D03_110.2	D03	mk12096_D03	mk12127_D03	26,385,562	30,628,951	4.24	92.11	5.76	−0.0231	0.0067	0.29	9.87	PD	NH
		qFW_D03_ck	D03	mk12142_D03	mk12152_D03	36,697,656	37,665,167	0.97	132.21	4.19	−0.0227	0.0241	1.06	8	D	NH
		qFW_D03_110.1	D03	mk15025	mk15027	5,017,089	5,704,231	0.69	81.91	6.05	−0.0219	−0.0093	0.42	7.24	PD	NH
		qFW_D03_150.1	D03	mk12142_D03	mk12154_D03	36,697,656	37,676,414	0.98	132.21	4.45	−0.0241	−0.014	0.58	5.46	PD	NH
		qFW_D03_150.2	D03	mk12140_D03	mk12154_D03	35,382,974	37,676,414	2.29	136.01	2.79	−0.0103	−0.0047	0.46	3.63	PD	NH
	SLW	qSLW_D03_110	D03	mk12142_D03	mk12152_D03	36,697,656	37,665,167	0.97	135.01	5.12	−0.0143	−0.0002	0.01	7.6	A	NH
		qSLW_D03_ck.1	D03	mk12140_D03	mk12154_D03	35,382,974	37,676,414	2.29	136.01	2.64	−0.0074	0.0168	2.27	4.83	OD	NH
		qSLW_D03_ck.2	D03	mk12158_D03	mk12165_D03	37,938,158	40,254,875	2.32	151.21	3.58	−0.0053	0.0223	4.21	4.28	OD	NH
		qSLW_D03_150	D03	mk12140_D03	mk12154_D03	35,382,974	37,676,414	2.29	135.01	3.27	−0.0117	−0.007	0.6	3.69	PD	NH
		qSLW_D03_ck.3	D03	mk12162_D03	mk12186_D03	40,254,814	42,120,184	1.87	157.61	2.81	−0.0034	0.0203	5.97	2.72	OD	NH
**6**	DLW	qDLW_D06_110	D06	mk13340_D06	mk13562_D06	11,196,129	23,393,584	12.2	59.21	4.21	−0.0008	0.002	2.5	5.85	OD	NH
		qDLW_D06_ck	D06	mk17780	mk13340_D06	982	11,196,129	11.2	59.21	3.25	−0.0006	0.002	3.33	4.29	OD	NH
	SLW	qSLW_D06_ck	D06	mk13340_D06	mk13562_D06	11,196,129	23,393,584	12.2	59.21	2.72	−0.0071	0.0146	2.06	4.4	OD	NH
		qSLW_D06_110	D06	mk13340_D06	mk13562_D06	11,196,129	23,393,584	12.2	59.21	3.14	−0.006	0.0215	3.58	3.79	OD	NH
**7**	FW	qFW_D08_150	D08	MulMa513_D08	mk16015_D08	54,937,777	59,655,801	4.72	200.81	2.66	0.0095	−0.0096	1.01	4.71	D	CCRI35
		qFW_D08_110	D08	MulMa512_D08	mk16022_D08	54,681,233	59,878,825	5.2	191.61	3.29	0.0171	0.0115	0.67	3.76	PD	CCRI35
		qFW_D08_110	D08	MulMa512_D08	MulMa517-m_D08	54,681,233	58,216,493	3.54	187.61	3.12	0.0087	0.0088	1.01	2.66	D	CCRI35
**8**	MDA	qMDA_D13_110	D13	mk18516_D13	mk18547_D13	41,759,681	48,359,057	6.6	1.31	2.78	−0.0021	0.0034	1.62	4.79	OD	NH
		qMDA_D13_150	D13	mk11123	mk7779	7462	58,175	0.05	80.21	2.93	0.001	0.0093	9.3	0.11	OD	CCRI35

LOD: logarithm of odds, 0 < Ae (additive effect) < 0.20; 0.21 < PD (partial dominance) < 0.80; 0.81 < D (dominance) < 1.20; OD (overdominance) > 1.20, |d/a| = De/Ae, GA: gene action, DPE: direction of phenotypic explanation.

## Supplementary Materials

Supplementary materials can be found at www.mdpi.com/1422-0067/18/12/2622/s1.

## Abbreviations

**Trait**	**Meaning**	**Unit**
MDA	Malondialdehyde	µM/g.FW
EC	Electric Conductivity	%
GR	Germination Rate	number/40 seeds
FW	Fresh Weight	g
SL	Stem Length	cm
LFW	Leaf Fresh Weight	g
SLW	Saturated Leaf Weight	g
DLW	Dry Leaf Weight	g
RWC	Related Water Content	mL
CHL	Chlorophyll	mg/g.FW
NH	Nan Dan Ba Di Da Hua	
CCRI35	Zhong35	
