# Supplementary material for: High-Density Linkage Map Construction and Mapping of Salt-Tolerant QTLs at Seedling Stage in Upland Cotton Using Genotyping by Sequencing (GBS)
Source: Int J Mol Sci. 2017 Dec 5;18(12):2622. doi: 10.3390/ijms18122622 (PMC5751225; doi:10.3390/ijms18122622)
Supplement: Supplementary file 1 [file ijms-18-02622-s001.zip › ijms-242441 final-supplementary/Figure S1 Frequency distribution of the 10 traits measured under 3 treatments during 2 years and combine analysis.pdf]

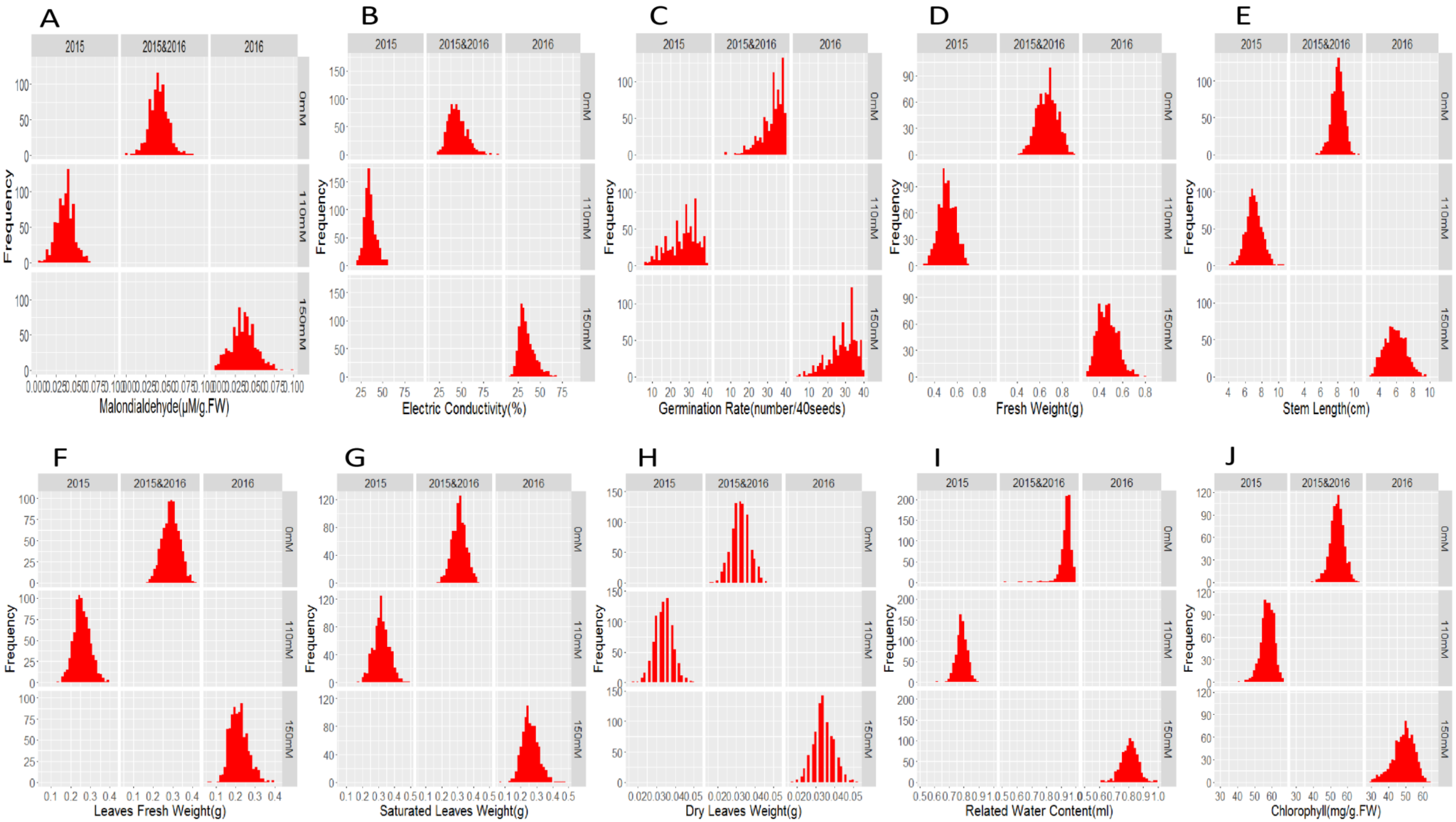


**Figure S1**: Frequency distribution of the 10 traits measured under 3 treatments during 2 years and combine analysis; **MDA**: Malondialdehyde; **EC**: Electric Conductivity; **GR**: Germination Rate; **FW**: Fresh Weight; **SL**: Stem Length; **LFW**: Leaves Fresh Weight; **SLW**: Saturated Leaves Weight; **DLW**: Dry Leaves Weight; **RWC:** Related Water Content and **CHL**: Chlorophyll. For the traits unit see **Figure 1**.
