# Supplementary material for: High-Density Linkage Map Construction and Mapping of Salt-Tolerant QTLs at Seedling Stage in Upland Cotton Using Genotyping by Sequencing (GBS)
Source: Int J Mol Sci. 2017 Dec 5;18(12):2622. doi: 10.3390/ijms18122622 (PMC5751225; doi:10.3390/ijms18122622)
Supplement: Supplementary file 1 [file ijms-18-02622-s001.zip › ijms-242441 final-supplementary/Figure S2 Total QTLs (66) found in this study, asterisk means consistent QTLs.pdf]

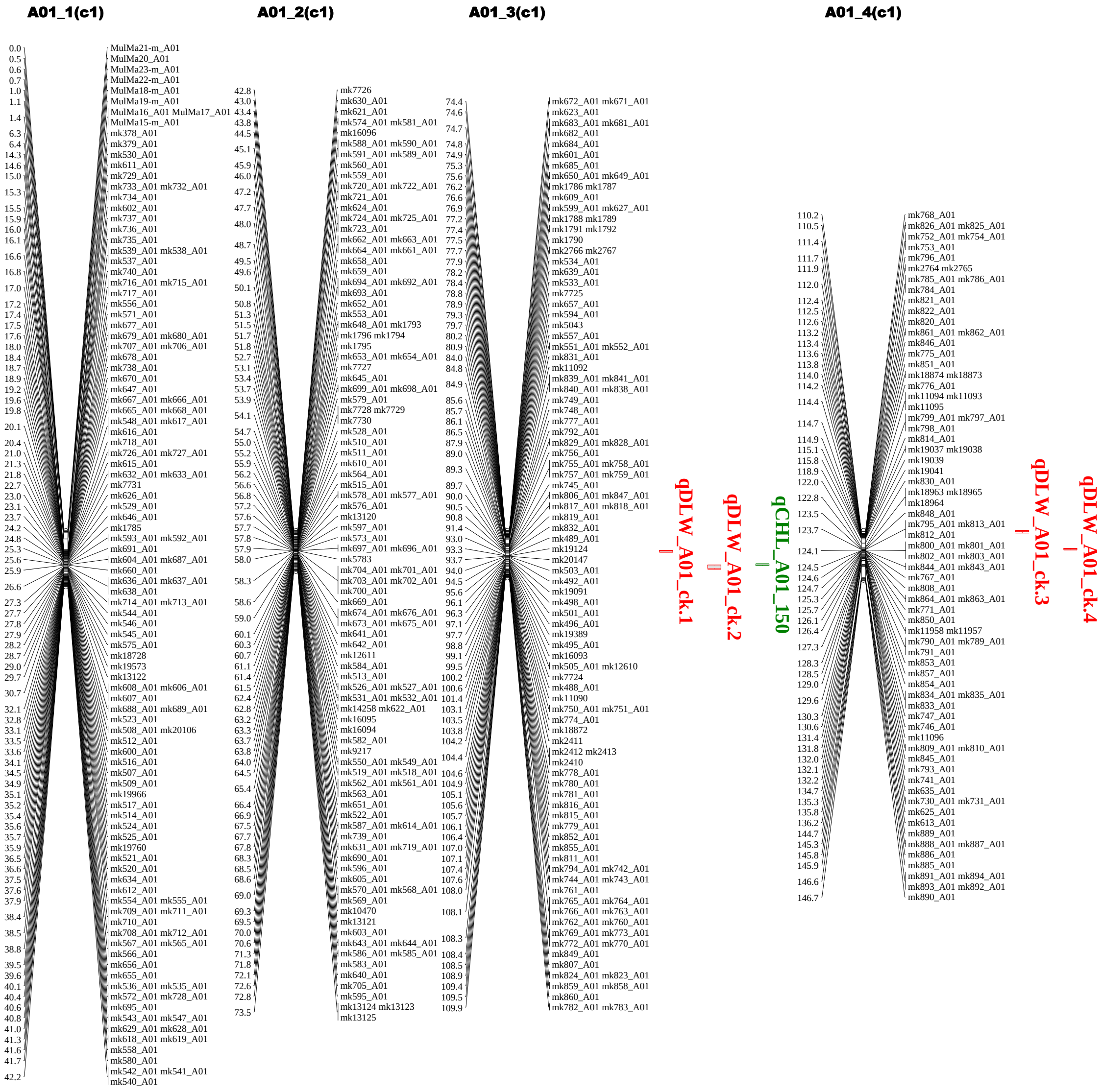

A01_1(c1)
A01_2(c1)
A01_3(c1)
A01_4(c1)
qDLW_A01_ck.1
qDLW_A01_ck.2
qCHL_A01_150
qDLW_A01_ck.3
qDLW_A01_ck.4

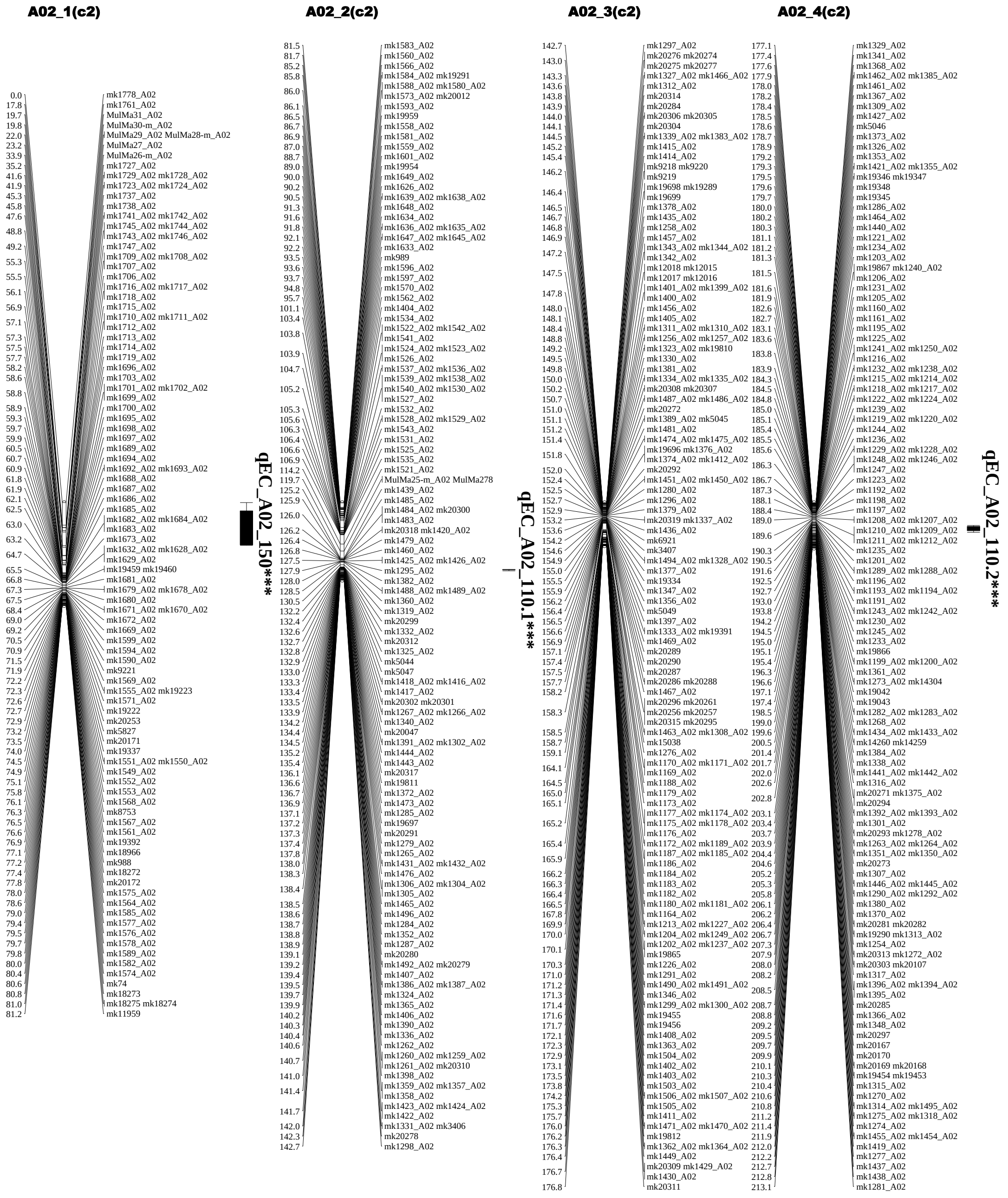

A02_1(c2)
A02_2(c2)
A02_3(c2)
A02_4(c2)
qEC_A02_150***
qEC_A02_110.1***
qEC_A02_110.2***

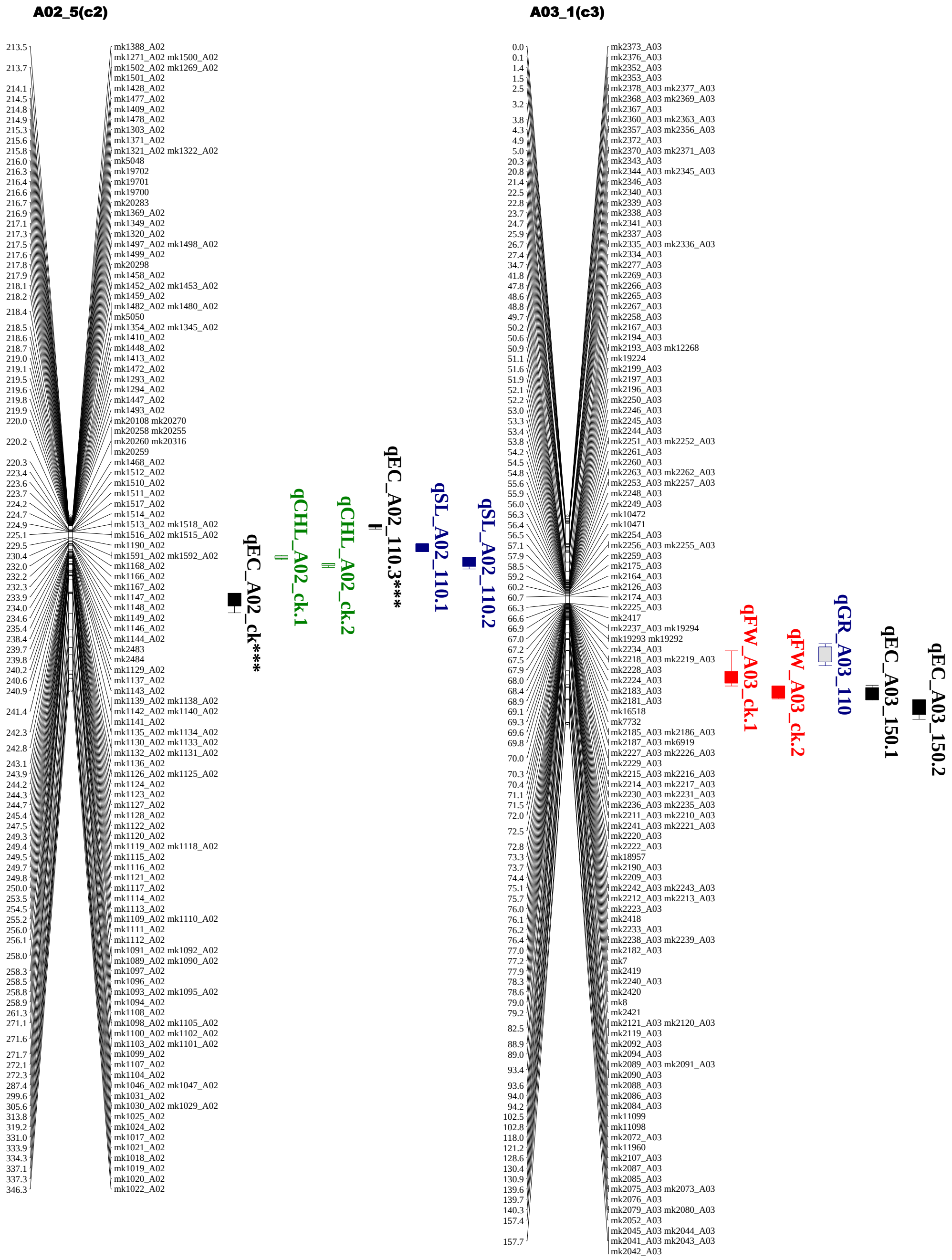
A02_5(c2)
A03_1(c3)
qEC_A02_ck***
qCHL_A02_ck.1
qCHL_A02_ck.2
qEC_A02_110.3***
qSL_A02_110.1
qSL_A02_110.2
qFW_A03_ck.1
qFW_A03_ck.2
qGR_A03_110
qEC_A03_150.1
qEC_A03_150.2

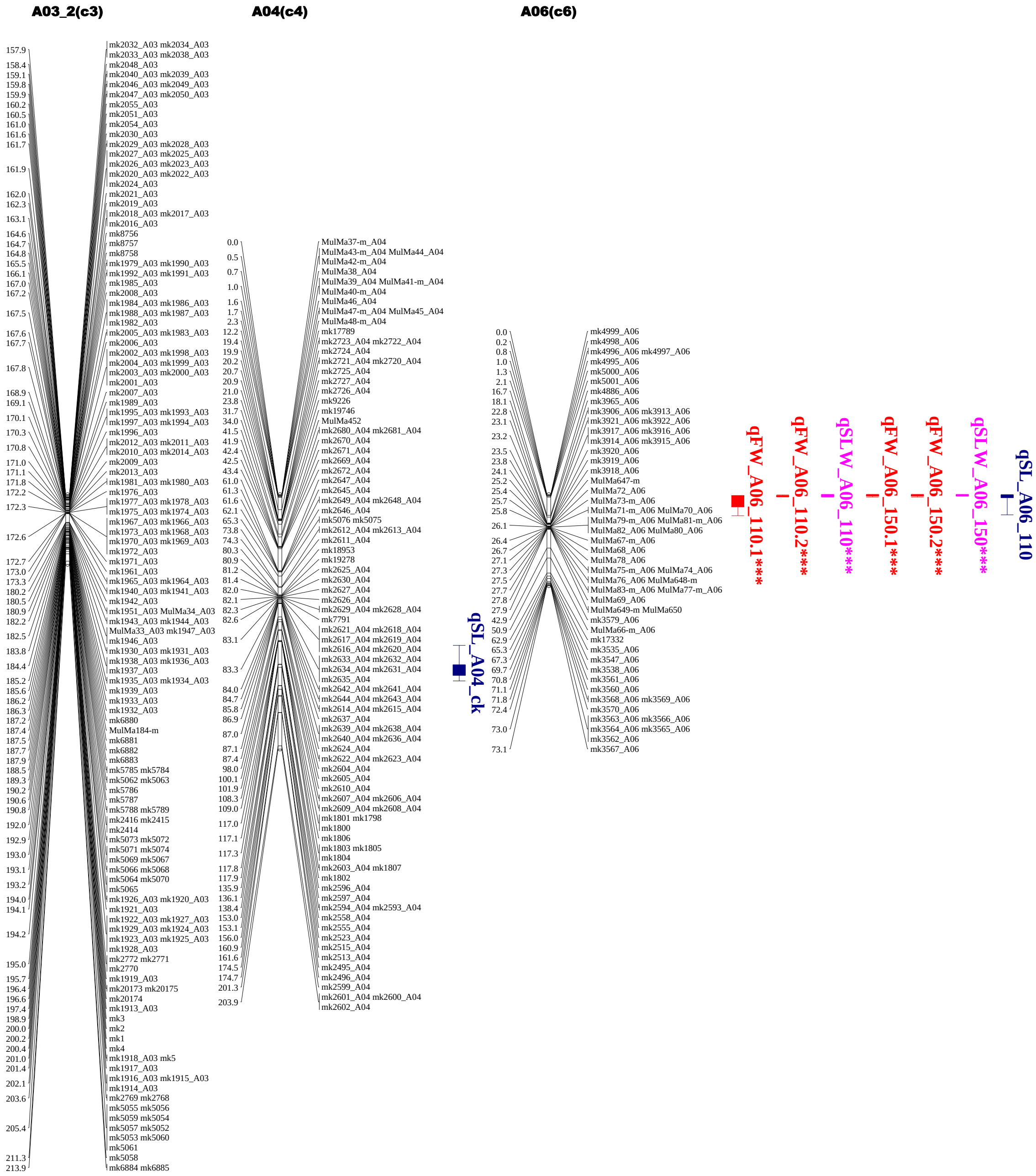

A03_2(c3)
A04(c4)
A06(c6)
qSL_A04_ck
qFW_A06_110.1***
qFW_A06_110.2***
qSLW_A06_110***
qFW_A06_150.1***
qFW_A06_150.2***
qSLW_A06_150***
qSL_A06_110

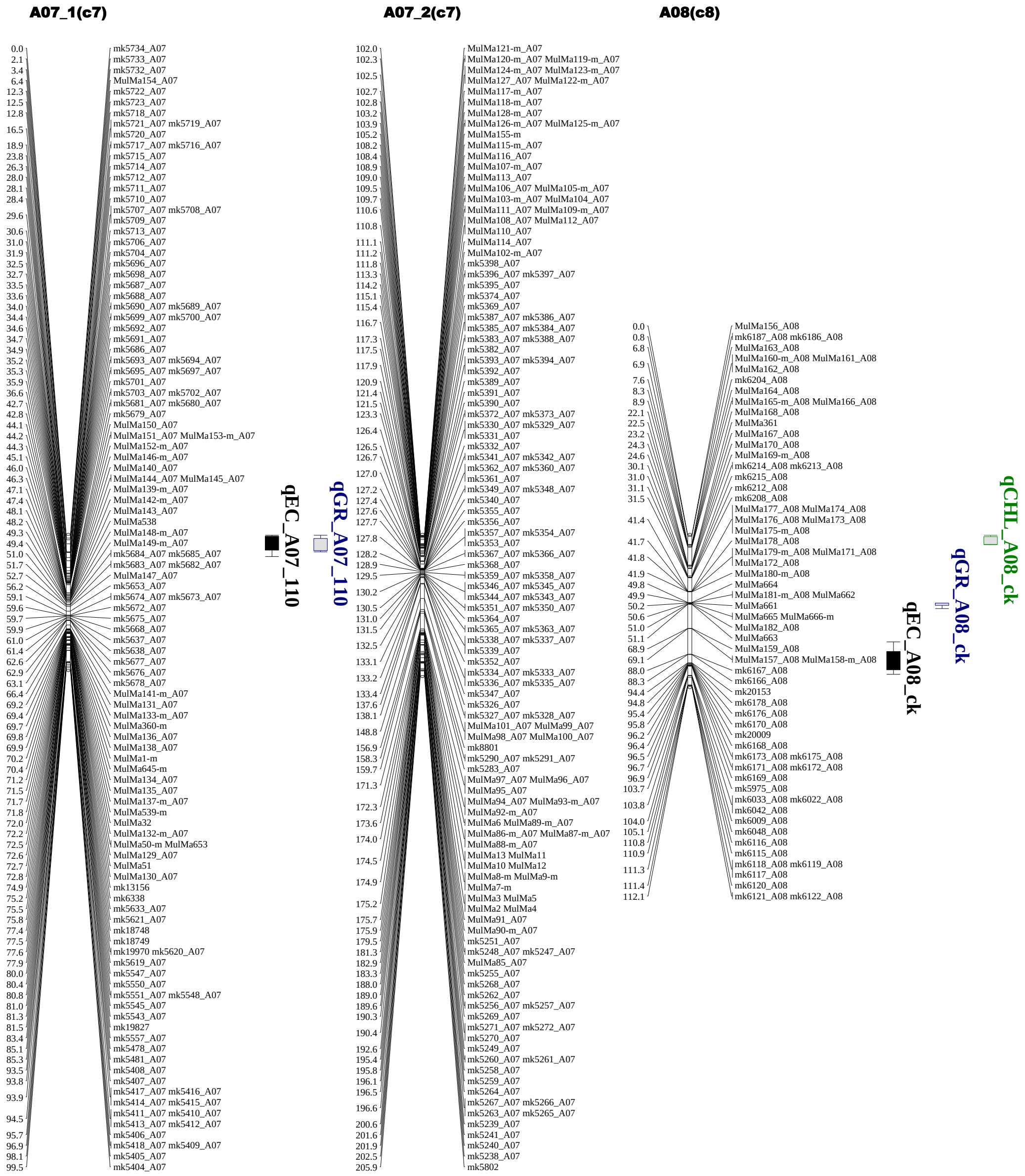
A07_1(c7)
A07_2(c7)
A08(c8)
0.0
2.1
3.4
6.4
12.3
12.5
12.8
16.5
18.9
23.8
26.3
28.0
28.1
28.4
29.6
30.6
31.0
31.9
32.5
32.7
33.5
33.6
34.0
34.4
34.6
34.7
34.9
35.2
35.3
35.9
36.6
42.7
42.8
44.1
44.2
44.3
45.1
46.0
46.3
47.1
47.4
48.1
48.2
49.3
49.4
51.0
51.7
52.7
56.2
59.1
59.6
59.7
59.9
61.0
61.4
62.6
62.9
63.1
66.4
69.2
69.4
69.7
69.8
69.9
70.2
70.4
71.2
71.5
71.7
71.8
72.0
72.2
72.5
72.6
72.7
72.8
74.9
75.2
75.5
75.8
77.4
77.5
77.6
77.9
80.0
80.4
80.8
81.0
81.3
81.5
83.4
85.1
85.3
93.5
93.8
93.9
94.5
95.7
96.9
98.1
99.5
mk5734_A07
mk5733_A07
mk5732_A07
MulMa154_A07
mk5722_A07
mk5723_A07
mk5718_A07
mk5721_A07 mk5719_A07
mk5720_A07
mk5717_A07 mk5716_A07
mk5715_A07
mk5714_A07
mk5712_A07
mk5711_A07
mk5710_A07
mk5707_A07 mk5708_A07
mk5709_A07
mk5713_A07
mk5706_A07
mk5704_A07
mk5696_A07
mk5698_A07
mk5687_A07
mk5688_A07
mk5690_A07 mk5689_A07
mk5699_A07 mk5700_A07
mk5692_A07
mk5691_A07
mk5686_A07
mk5693_A07 mk5694_A07
mk5695_A07 mk5697_A07
mk5701_A07
mk5703_A07 mk5702_A07
mk5681_A07 mk5680_A07
mk5679_A07
MulMa150_A07
MulMa151_A07 MulMa153-m_A07
MulMa152-m_A07
MulMa146-m_A07
MulMa140_A07
MulMa144_A07 MulMa145_A07
MulMa139-m_A07
MulMa142-m_A07
MulMa143_A07
MulMa538
MulMa148-m_A07
MulMa149-m_A07
mk5684_A07 mk5685_A07
mk5683_A07 mk5682_A07
MulMa147_A07
mk5653_A07
mk5674_A07 mk5673_A07
mk5672_A07
mk5675_A07
mk5668_A07
mk5637_A07
mk5638_A07
mk5677_A07
mk5676_A07
mk5678_A07
MulMa141-m_A07
MulMa131_A07
MulMa133-m_A07
MulMa360-m
MulMa136_A07
MulMa138_A07
MulMa1-m
MulMa645-m
MulMa134_A07
MulMa135_A07
MulMa137-m_A07
MulMa539-m
MulMa32
MulMa132-m_A07
MulMa50-m MulMa653
MulMa129_A07
MulMa51
MulMa130_A07
mk13156
mk6338
mk5633_A07
mk5621_A07
mk18748
mk18749
mk19970 mk5620_A07
mk5619_A07
mk5547_A07
mk5550_A07
mk5551_A07 mk5548_A07
mk5545_A07
mk5543_A07
mk19827
mk5557_A07
mk5478_A07
mk5481_A07
mk5408_A07
mk5407_A07
mk5417_A07 mk5416_A07
mk5414_A07 mk5415_A07
mk5411_A07 mk5410_A07
mk5413_A07 mk5412_A07
mk5406_A07
mk5418_A07 mk5409_A07
mk5405_A07
mk5404_A07
qEC_A07_110
qGR_A07_110
102.0
102.3
102.5
102.7
102.8
103.2
103.9
105.2
108.2
108.4
108.9
109.0
109.5
109.7
110.6
110.8
111.1
111.2
111.8
113.3
114.2
115.1
115.4
116.7
117.3
117.5
117.9
120.9
121.4
121.5
123.3
126.4
126.5
126.7
127.0
127.2
127.4
127.6
127.7
127.8
128.2
128.9
129.5
130.2
130.5
131.0
131.5
132.5
133.1
133.2
133.4
137.6
138.1
148.8
156.9
158.3
159.7
171.3
172.3
173.6
174.0
174.5
174.9
175.2
175.7
175.9
179.5
181.3
182.9
183.3
188.0
189.0
189.6
190.3
190.4
192.6
195.4
195.8
196.1
196.5
196.6
200.6
201.6
201.9
202.5
205.9
MulMa121-m_A07
MulMa120-m_A07 MulMa119-m_A07
MulMa124-m_A07 MulMa123-m_A07
MulMa127_A07 MulMa122-m_A07
MulMa117-m_A07
MulMa118-m_A07
MulMa128-m_A07
MulMa126-m_A07 MulMa125-m_A07
MulMa155-m
MulMa115-m_A07
MulMa116_A07
MulMa107-m_A07
MulMa113_A07
MulMa106_A07 MulMa105-m_A07
MulMa103-m_A07 MulMa104_A07
MulMa111_A07 MulMa109-m_A07
MulMa108_A07 MulMa112_A07
MulMa110_A07
MulMa114_A07
MulMa102-m_A07
mk5398_A07
mk5396_A07 mk5397_A07
mk5395_A07
mk5374_A07
mk5369_A07
mk5387_A07 mk5386_A07
mk5385_A07 mk5384_A07
mk5383_A07 mk5388_A07
mk5382_A07
mk5393_A07 mk5394_A07
mk5392_A07
mk5389_A07
mk5391_A07
mk5390_A07
mk5372_A07 mk5373_A07
mk5330_A07 mk5329_A07
mk5331_A07
mk5332_A07
mk5341_A07 mk5342_A07
mk5362_A07 mk5360_A07
mk5361_A07
mk5349_A07 mk5348_A07
mk5340_A07
mk5355_A07
mk5356_A07
mk5357_A07 mk5354_A07
mk5353_A07
mk5367_A07 mk5366_A07
mk5368_A07
mk5359_A07 mk5358_A07
mk5346_A07 mk5345_A07
mk5344_A07 mk5343_A07
mk5351_A07 mk5350_A07
mk5364_A07
mk5365_A07 mk5363_A07
mk5338_A07 mk5337_A07
mk5339_A07
mk5352_A07
mk5334_A07 mk5333_A07
mk5336_A07 mk5335_A07
mk5347_A07
mk5326_A07
mk5327_A07 mk5328_A07
MulMa101_A07 MulMa99_A07
MulMa98_A07 MulMa100_A07
mk8801
mk5290_A07 mk5291_A07
mk5283_A07
MulMa97_A07 MulMa96_A07
MulMa95_A07
MulMa94_A07 MulMa93-m_A07
MulMa92-m_A07
MulMa6 MulMa89-m_A07
MulMa86-m_A07 MulMa87-m_A07
MulMa88-m_A07
MulMa13 MulMa11
MulMa10 MulMa12
MulMa8-m MulMa9-m
MulMa7-m
MulMa3 MulMa5
MulMa2 MulMa4
MulMa91_A07
MulMa90-m_A07
mk5251_A07
mk5248_A07 mk5247_A07
MulMa85_A07
mk5255_A07
mk5268_A07
mk5262_A07
mk5256_A07 mk5257_A07
mk5269_A07
mk5271_A07 mk5272_A07
mk5270_A07
mk5249_A07
mk5260_A07 mk5261_A07
mk5258_A07
mk5259_A07
mk5264_A07
mk5267_A07 mk5266_A07
mk5263_A07 mk5265_A07
mk5239_A07
mk5241_A07
mk5240_A07
mk5238_A07
mk5802
0.0
0.8
6.8
6.9
7.6
8.3
8.9
22.1
22.5
23.2
24.3
24.6
30.1
31.0
31.1
31.5
41.4
41.7
41.8
41.9
49.8
49.9
50.2
50.6
51.0
51.1
68.9
69.1
88.0
88.3
94.4
94.8
95.4
95.8
96.2
96.4
96.5
96.7
96.9
103.7
103.8
104.0
105.1
110.8
110.9
111.3
111.4
112.1
MulMa156_A08
mk6187_A08 mk6186_A08
MulMa163_A08
MulMa160-m_A08 MulMa161_A08
MulMa162_A08
mk6204_A08
MulMa164_A08
MulMa165-m_A08 MulMa166_A08
MulMa168_A08
MulMa361
MulMa167_A08
MulMa170_A08
MulMa169-m_A08
mk6214_A08 mk6213_A08
mk6215_A08
mk6212_A08
mk6208_A08
MulMa177_A08 MulMa174_A08
MulMa176_A08 MulMa173_A08
MulMa175-m_A08
MulMa178_A08
MulMa179-m_A08 MulMa171_A08
MulMa172_A08
MulMa180-m_A08
MulMa664
MulMa181-m_A08 MulMa662
MulMa661
MulMa665 MulMa666-m
MulMa182_A08
MulMa663
MulMa159_A08
MulMa157_A08 MulMa158-m_A08
mk6167_A08
mk6166_A08
mk20153
mk6178_A08
mk6176_A08
mk6170_A08
mk20009
mk6168_A08
mk6173_A08 mk6175_A08
mk6171_A08 mk6172_A08
mk6169_A08
mk5975_A08
mk6033_A08 mk6022_A08
mk6042_A08
mk6009_A08
mk6048_A08
mk6116_A08
mk6115_A08
mk6118_A08 mk6119_A08
mk6117_A08
mk6120_A08
mk6121_A08 mk6122_A08
qCHL_A08_ck
qGR_A08_ck
qEC_A08_ck

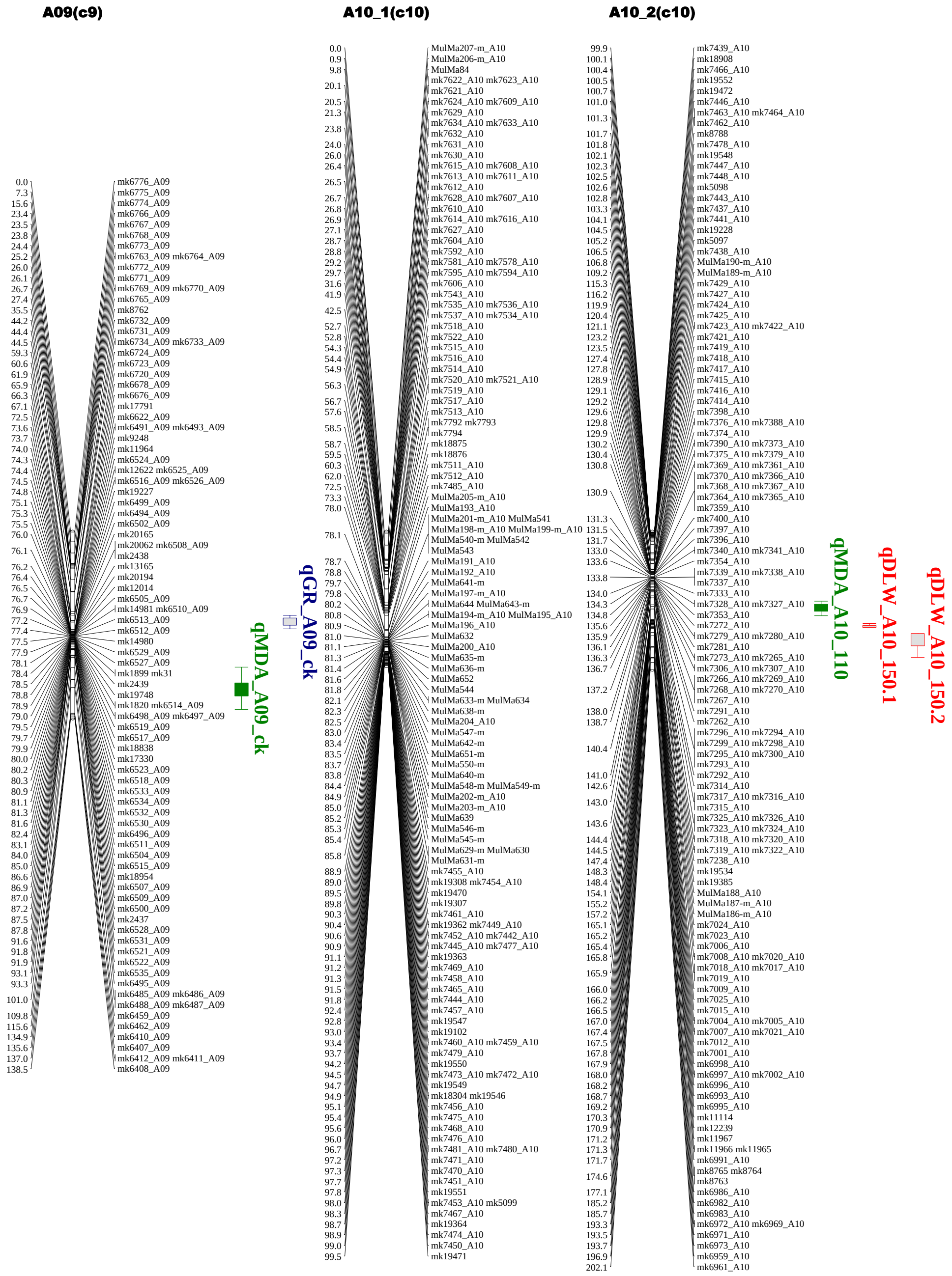

A09(c9)
A10_1(c10)
A10_2(c10)
qGR_A09_ck
qMDA_A09_ck
qMDA_A10_110
qDLW_A10_150.1
qDLW_A10_150.2

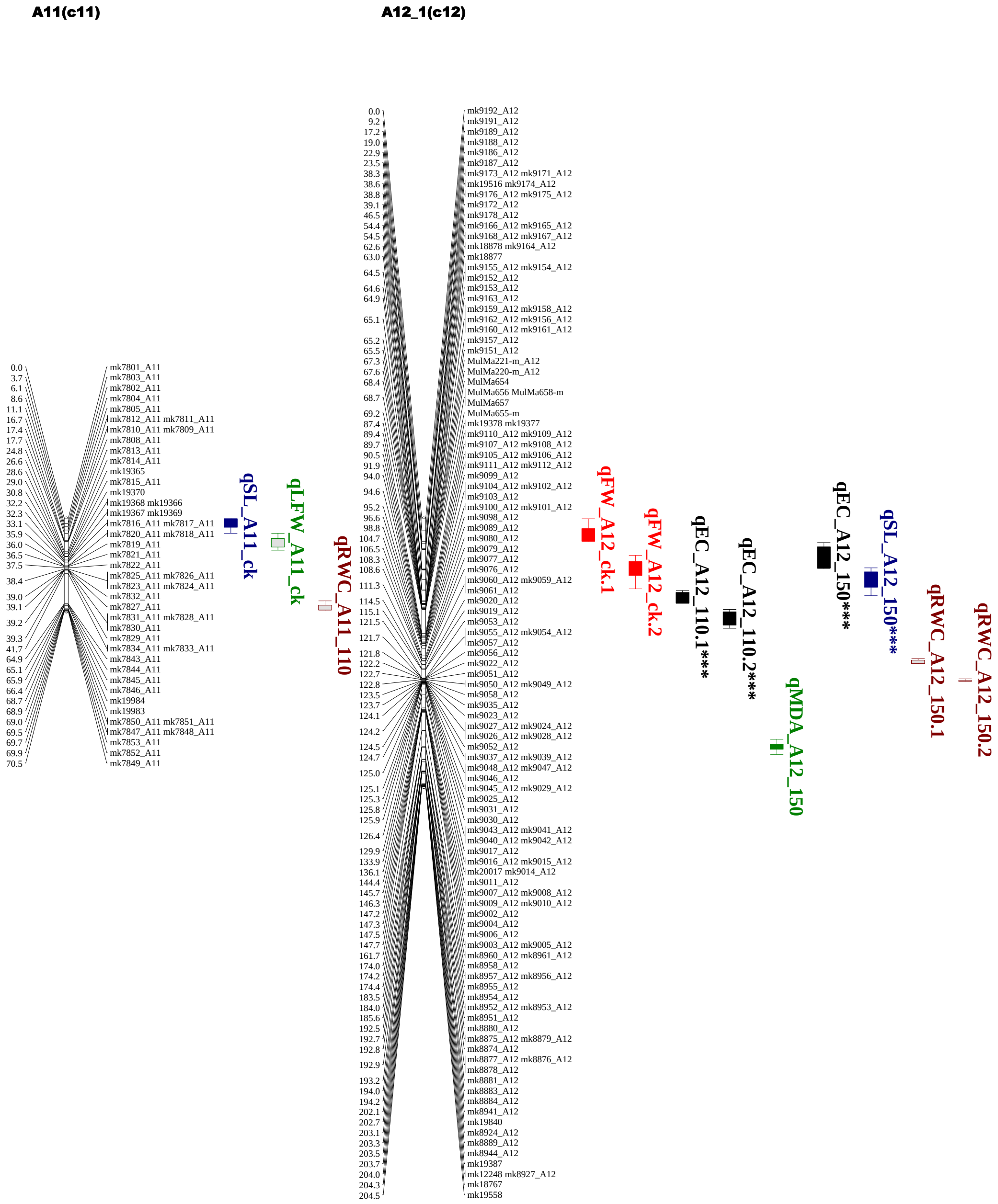

A11(c11)
A12_1(c12)
0.0 mk7801_A11
3.7 mk7803_A11
6.1 mk7802_A11
8.6 mk7804_A11
11.1 mk7805_A11
16.7 mk7812_A11 mk7811_A11
17.4 mk7810_A11 mk7809_A11
17.7 mk7808_A11
24.8 mk7813_A11
26.6 mk7814_A11
28.6 mk19365
29.0 mk7815_A11
30.8 mk19370
32.2 mk19368 mk19366
32.3 mk19367 mk19369
33.1 mk7816_A11 mk7817_A11
35.9 mk7820_A11 mk7818_A11
36.0 mk7819_A11
36.5 mk7821_A11
37.5 mk7822_A11
38.4 mk7825_A11 mk7826_A11
mk7823_A11 mk7824_A11
39.0 mk7832_A11
39.1 mk7827_A11
39.2 mk7831_A11 mk7828_A11
mk7830_A11
39.3 mk7829_A11
41.7 mk7834_A11 mk7833_A11
64.9 mk7843_A11
65.1 mk7844_A11
65.9 mk7845_A11
66.4 mk7846_A11
68.7 mk19984
68.9 mk19983
69.0 mk7850_A11 mk7851_A11
69.5 mk7847_A11 mk7848_A11
69.7 mk7853_A11
69.9 mk7852_A11
70.5 mk7849_A11
qSL_A11_ck
qLFW_A11_ck
qRWC_A11_110
0.0 mk9192_A12
9.2 mk9191_A12
17.2 mk9189_A12
19.0 mk9188_A12
22.9 mk9186_A12
23.5 mk9187_A12
38.3 mk9173_A12 mk9171_A12
38.6 mk19516 mk9174_A12
38.8 mk9176_A12 mk9175_A12
39.1 mk9172_A12
46.5 mk9178_A12
54.4 mk9166_A12 mk9165_A12
54.5 mk9168_A12 mk9167_A12
62.6 mk18878 mk9164_A12
63.0 mk18877
64.5 mk9155_A12 mk9154_A12
64.6 mk9152_A12
64.9 mk9153_A12
mk9163_A12
65.1 mk9159_A12 mk9158_A12
mk9162_A12 mk9156_A12
mk9160_A12 mk9161_A12
65.2 mk9157_A12
65.5 mk9151_A12
67.3 MulMa221-m_A12
67.6 MulMa220-m_A12
68.4 MulMa654
68.7 MulMa656 MulMa658-m
MulMa657
69.2 MulMa655-m
87.4 mk19378 mk19377
89.4 mk9110_A12 mk9109_A12
89.7 mk9107_A12 mk9108_A12
90.5 mk9105_A12 mk9106_A12
91.9 mk9111_A12 mk9112_A12
94.0 mk9099_A12
94.6 mk9104_A12 mk9102_A12
mk9103_A12
95.2 mk9100_A12 mk9101_A12
96.6 mk9098_A12
98.8 mk9089_A12
104.7 mk9080_A12
106.5 mk9079_A12
108.3 mk9077_A12
108.6 mk9076_A12
111.3 mk9060_A12 mk9059_A12
mk9061_A12
114.5 mk9020_A12
115.1 mk9019_A12
121.5 mk9053_A12
121.7 mk9055_A12 mk9054_A12
mk9057_A12
121.8 mk9056_A12
122.2 mk9022_A12
122.7 mk9051_A12
122.8 mk9050_A12 mk9049_A12
123.5 mk9058_A12
123.7 mk9035_A12
124.1 mk9023_A12
124.2 mk9027_A12 mk9024_A12
mk9026_A12 mk9028_A12
124.5 mk9052_A12
124.7 mk9037_A12 mk9039_A12
125.0 mk9048_A12 mk9047_A12
mk9046_A12
125.1 mk9045_A12 mk9029_A12
125.3 mk9025_A12
125.8 mk9031_A12
125.9 mk9030_A12
126.4 mk9043_A12 mk9041_A12
mk9040_A12 mk9042_A12
129.9 mk9017_A12
133.9 mk9016_A12 mk9015_A12
136.1 mk20017 mk9014_A12
144.4 mk9011_A12
145.7 mk9007_A12 mk9008_A12
146.3 mk9009_A12 mk9010_A12
147.2 mk9002_A12
147.3 mk9004_A12
147.5 mk9006_A12
147.7 mk9003_A12 mk9005_A12
161.7 mk8960_A12 mk8961_A12
174.0 mk8958_A12
174.2 mk8957_A12 mk8956_A12
174.4 mk8955_A12
183.5 mk8954_A12
184.0 mk8952_A12 mk8953_A12
185.6 mk8951_A12
192.5 mk8880_A12
192.7 mk8875_A12 mk8879_A12
192.8 mk8874_A12
192.9 mk8877_A12 mk8876_A12
mk8878_A12
193.2 mk8881_A12
194.0 mk8883_A12
194.2 mk8884_A12
202.1 mk8941_A12
202.7 mk19840
203.1 mk8924_A12
203.3 mk8889_A12
203.5 mk8944_A12
203.7 mk19387
204.0 mk12248 mk8927_A12
204.3 mk18767
204.5 mk19558
qFW_A12_ck.1
qFW_A12_ck.2
qEC_A12_110.1***
qEC_A12_110.2***
qMDA_A12_150
qEC_A12_150***
qSL_A12_150***
qRWC_A12_150.1
qRWC_A12_150.2

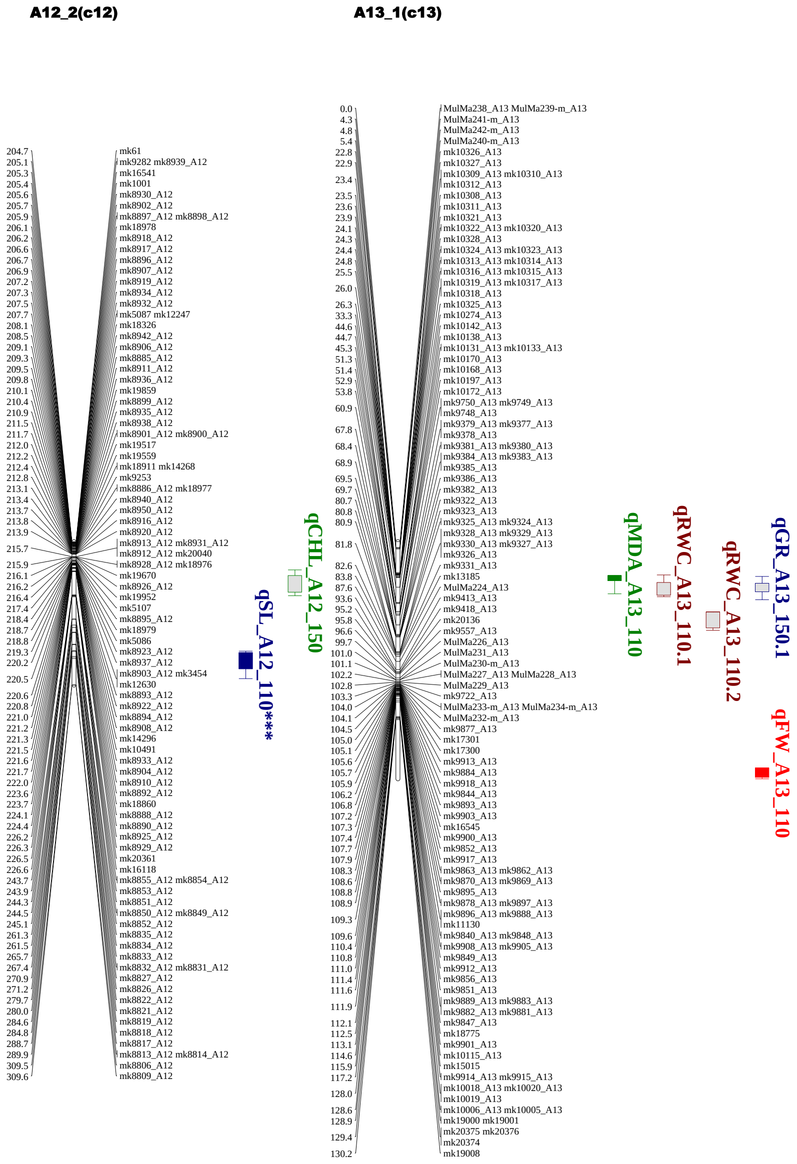
A12_2(c12)
A13_1(c13)
204.7
205.1
205.3
205.4
205.6
205.7
205.9
206.1
206.2
206.6
206.7
206.9
207.2
207.3
207.5
207.7
208.1
208.5
209.1
209.3
209.5
209.8
210.1
210.4
210.9
211.5
211.7
212.0
212.2
212.4
212.8
213.1
213.4
213.7
213.8
213.9
215.7
215.9
216.1
216.2
216.4
217.4
218.4
218.7
218.8
219.3
220.2
220.5
220.6
220.8
221.0
221.2
221.3
221.5
221.6
221.7
222.0
223.6
223.7
224.1
224.4
226.2
226.3
226.5
226.6
243.7
243.9
244.3
244.5
245.1
261.3
261.5
265.7
267.4
270.9
271.2
279.7
280.0
284.6
284.8
288.7
289.9
309.5
309.6
mk61
mk9282 mk8939_A12
mk16541
mk1001
mk8930_A12
mk8902_A12
mk8897_A12 mk8898_A12
mk18978
mk8918_A12
mk8917_A12
mk8896_A12
mk8907_A12
mk8919_A12
mk8934_A12
mk8932_A12
mk5087 mk12247
mk18326
mk8942_A12
mk8906_A12
mk8885_A12
mk8911_A12
mk8936_A12
mk19859
mk8899_A12
mk8935_A12
mk8938_A12
mk8901_A12 mk8900_A12
mk19517
mk19559
mk18911 mk14268
mk9253
mk8886_A12 mk18977
mk8940_A12
mk8950_A12
mk8916_A12
mk8920_A12
mk8913_A12 mk8931_A12
mk8912_A12 mk20040
mk8928_A12 mk18976
mk19670
mk8926_A12
mk19952
mk5107
mk8895_A12
mk18979
mk5086
mk8923_A12
mk8937_A12
mk8903_A12 mk3454
mk12630
mk8893_A12
mk8922_A12
mk8894_A12
mk8908_A12
mk14296
mk10491
mk8933_A12
mk8904_A12
mk8910_A12
mk8892_A12
mk18860
mk8888_A12
mk8890_A12
mk8925_A12
mk8929_A12
mk20361
mk16118
mk8855_A12 mk8854_A12
mk8853_A12
mk8851_A12
mk8850_A12 mk8849_A12
mk8852_A12
mk8835_A12
mk8834_A12
mk8833_A12
mk8832_A12 mk8831_A12
mk8827_A12
mk8826_A12
mk8822_A12
mk8821_A12
mk8819_A12
mk8818_A12
mk8817_A12
mk8813_A12 mk8814_A12
mk8806_A12
mk8809_A12
qSL_A12_110***
qCHL_A12_150
0.0
4.3
4.8
5.4
22.8
22.9
23.4
23.5
23.6
23.9
24.1
24.3
24.4
24.8
25.5
26.0
26.3
33.3
44.6
44.7
45.3
51.3
51.4
52.9
53.8
60.9
67.8
68.4
68.9
69.5
69.7
80.7
80.8
80.9
81.8
82.6
83.8
87.6
93.6
95.2
95.8
96.6
99.7
101.0
101.1
102.2
102.8
103.3
104.0
104.1
104.5
105.0
105.1
105.6
105.7
105.9
106.2
106.8
107.2
107.3
107.4
107.7
107.9
108.3
108.6
108.8
108.9
109.3
109.6
110.4
110.8
111.0
111.4
111.6
111.9
112.1
112.5
113.1
114.6
115.9
117.2
128.0
128.6
128.9
129.4
130.2
MulMa238_A13 MulMa239-m_A13
MulMa241-m_A13
MulMa242-m_A13
MulMa240-m_A13
mk10326_A13
mk10327_A13
mk10309_A13 mk10310_A13
mk10312_A13
mk10308_A13
mk10311_A13
mk10321_A13
mk10322_A13 mk10320_A13
mk10328_A13
mk10324_A13 mk10323_A13
mk10313_A13 mk10314_A13
mk10316_A13 mk10315_A13
mk10319_A13 mk10317_A13
mk10318_A13
mk10325_A13
mk10274_A13
mk10142_A13
mk10138_A13
mk10131_A13 mk10133_A13
mk10170_A13
mk10168_A13
mk10197_A13
mk10172_A13
mk9750_A13 mk9749_A13
mk9748_A13
mk9379_A13 mk9377_A13
mk9378_A13
mk9381_A13 mk9380_A13
mk9384_A13 mk9383_A13
mk9385_A13
mk9386_A13
mk9382_A13
mk9322_A13
mk9323_A13
mk9325_A13 mk9324_A13
mk9328_A13 mk9329_A13
mk9330_A13 mk9327_A13
mk9326_A13
mk9331_A13
mk13185
MulMa224_A13
mk9413_A13
mk9418_A13
mk20136
mk9557_A13
MulMa226_A13
MulMa231_A13
MulMa230-m_A13
MulMa227_A13 MulMa228_A13
MulMa229_A13
mk9722_A13
MulMa233-m_A13 MulMa234-m_A13
MulMa232-m_A13
mk9877_A13
mk17301
mk17300
mk9913_A13
mk9884_A13
mk9918_A13
mk9844_A13
mk9893_A13
mk9903_A13
mk16545
mk9900_A13
mk9852_A13
mk9917_A13
mk9863_A13 mk9862_A13
mk9870_A13 mk9869_A13
mk9895_A13
mk9878_A13 mk9897_A13
mk9896_A13 mk9888_A13
mk11130
mk9840_A13 mk9848_A13
mk9908_A13 mk9905_A13
mk9849_A13
mk9912_A13
mk9856_A13
mk9851_A13
mk9889_A13 mk9883_A13
mk9882_A13 mk9881_A13
mk9847_A13
mk18775
mk9901_A13
mk10115_A13
mk15015
mk9914_A13 mk9915_A13
mk10018_A13 mk10020_A13
mk10019_A13
mk10006_A13 mk10005_A13
mk19000 mk19001
mk20375 mk20376
mk20374
mk19008
qMDA_A13_110
qRWC_A13_110.1
qRWC_A13_110.2
qGR_A13_150.1
qFW_A13_110

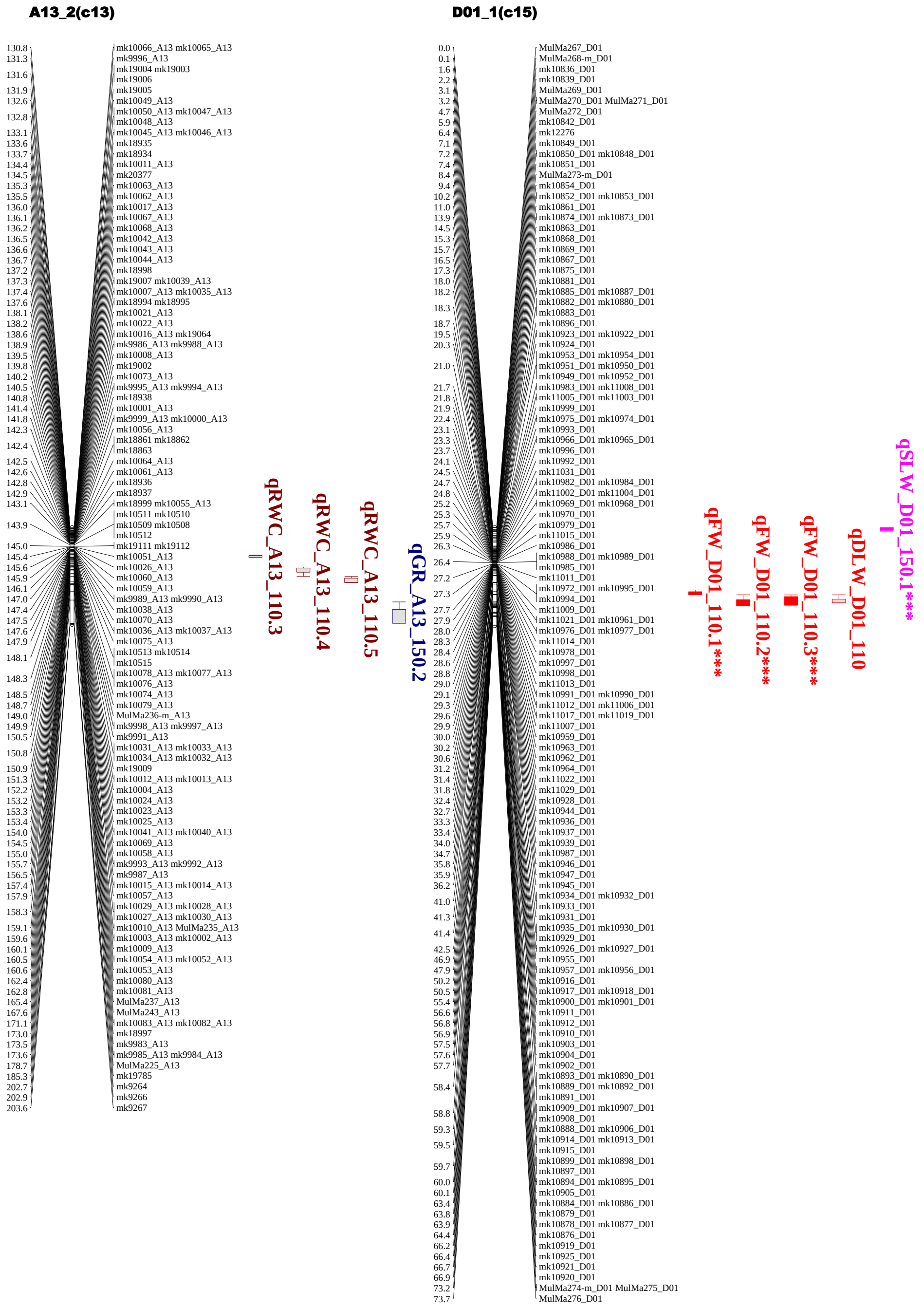
A13_2(c13)
D01_1(c15)
qRWC_A13_110.3
qRWC_A13_110.4
qRWC_A13_110.5
qGR_A13_150.2
qFW_D01_110.1***
qFW_D01_110.2***
qFW_D01_110.3***
qDLW_D01_110
qSLW_D01_150.1***

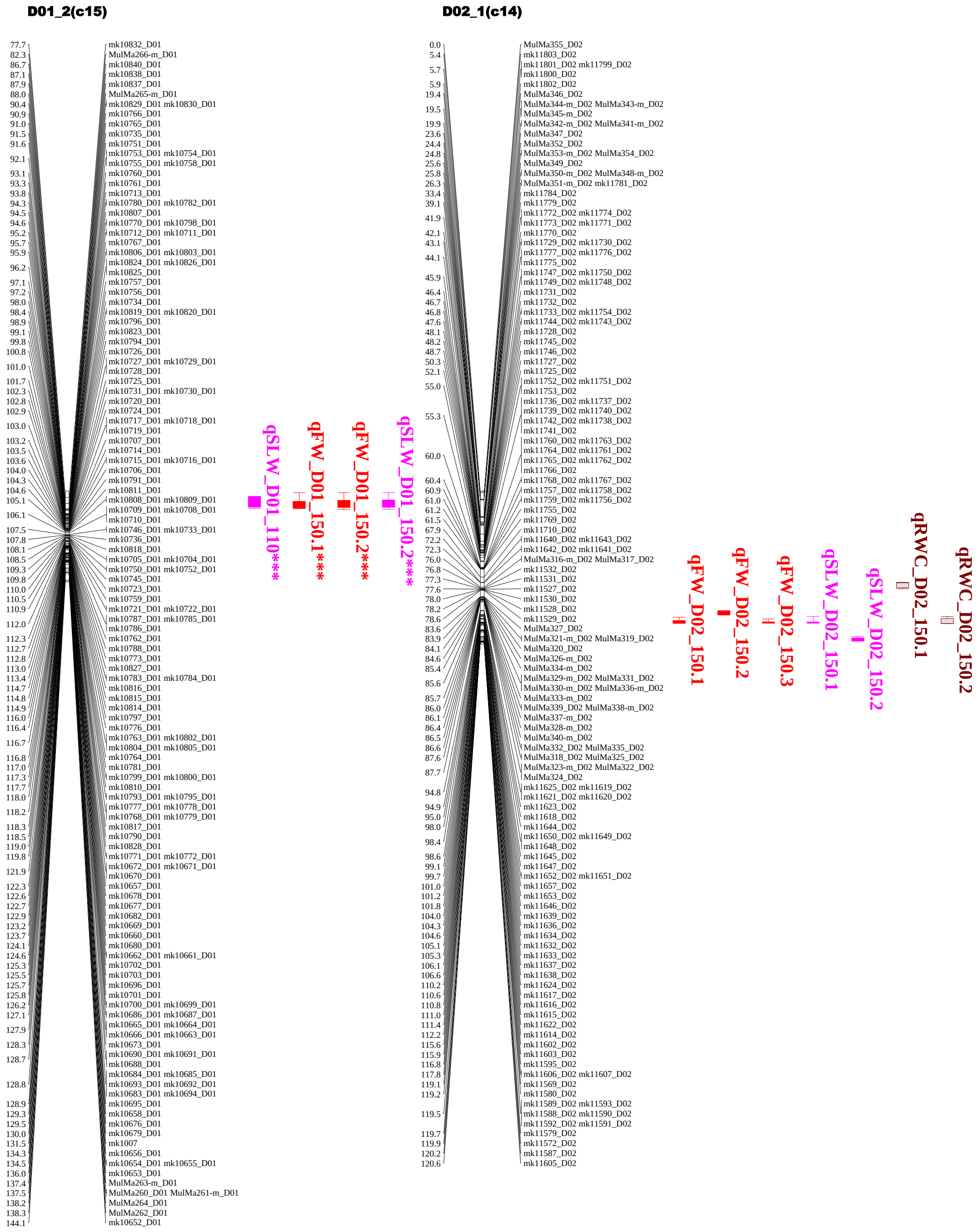
D01_2(c15)
D02_1(c14)
qSLW_D01_110***
qFW_D01_150.1***
qFW_D01_150.2***
qSLW_D01_150.2***
qFW_D02_150.1
qFW_D02_150.2
qFW_D02_150.3
qSLW_D02_150.1
qSLW_D02_150.2
qRWC_D02_150.1
qRWC_D02_150.2

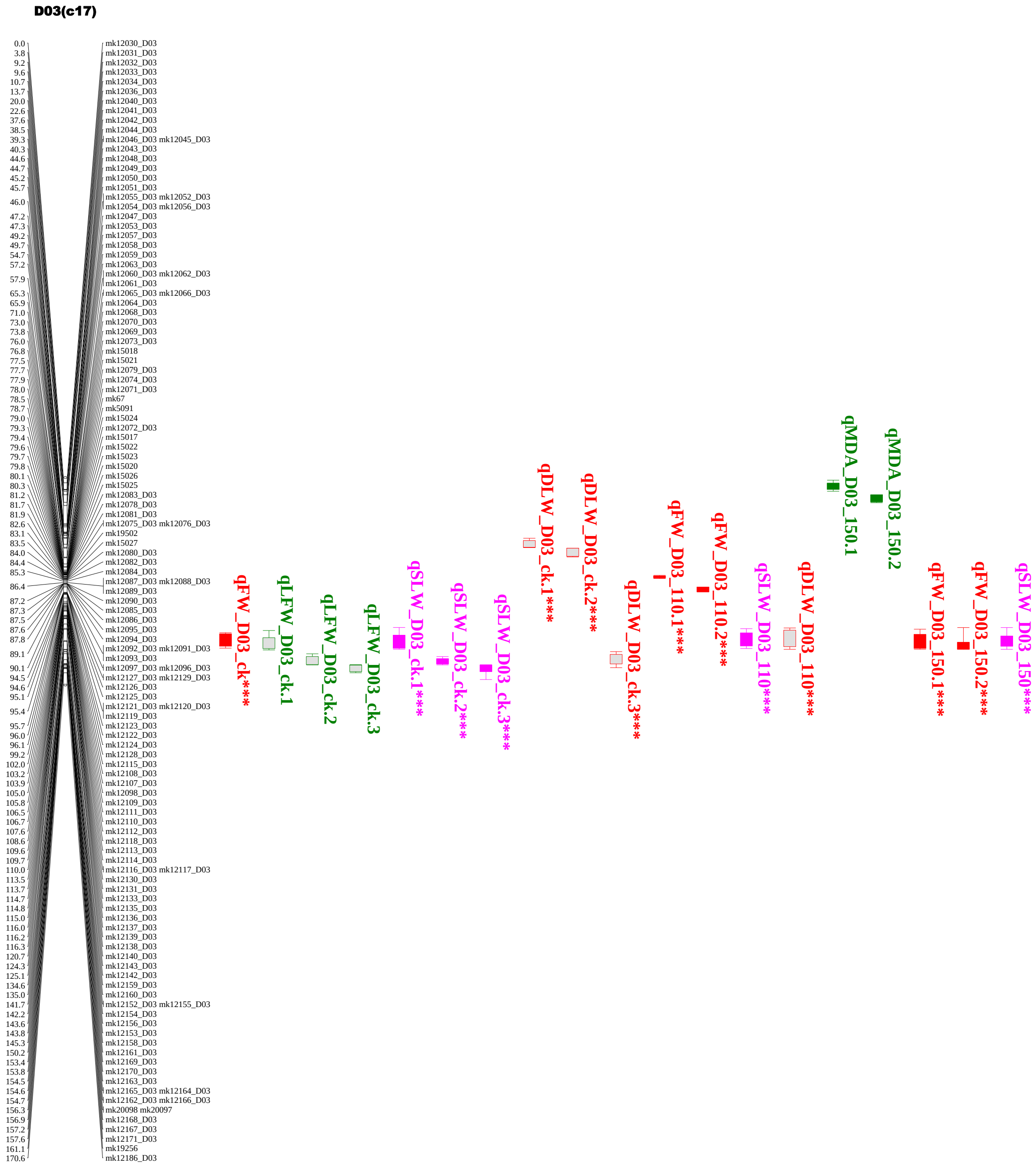
D03(c17)
0.0 mk12030_D03
3.8 mk12031_D03
9.2 mk12032_D03
9.6 mk12033_D03
10.7 mk12034_D03
13.7 mk12036_D03
20.0 mk12040_D03
22.6 mk12041_D03
37.6 mk12042_D03
38.5 mk12044_D03
39.3 mk12046_D03 mk12045_D03
40.3 mk12043_D03
44.6 mk12048_D03
44.7 mk12049_D03
45.2 mk12050_D03
45.7 mk12051_D03
46.0 mk12055_D03 mk12052_D03
mk12054_D03 mk12056_D03
47.2 mk12047_D03
47.3 mk12053_D03
49.2 mk12057_D03
49.7 mk12058_D03
54.7 mk12059_D03
57.2 mk12063_D03
57.9 mk12060_D03 mk12062_D03
mk12061_D03
65.3 mk12065_D03 mk12066_D03
65.9 mk12064_D03
71.0 mk12068_D03
73.0 mk12070_D03
73.8 mk12069_D03
76.0 mk12073_D03
76.8 mk15018
77.5 mk15021
77.7 mk12079_D03
77.9 mk12074_D03
78.0 mk12071_D03
78.5 mk67
78.7 mk5091
79.0 mk15024
79.3 mk12072_D03
79.4 mk15017
79.6 mk15022
79.7 mk15023
79.8 mk15020
80.1 mk15026
80.3 mk15025
81.2 mk12083_D03
81.7 mk12078_D03
81.9 mk12081_D03
82.6 mk12075_D03 mk12076_D03
83.1 mk19502
83.5 mk15027
84.0 mk12080_D03
84.4 mk12082_D03
85.3 mk12084_D03
86.4 mk12087_D03 mk12088_D03
mk12089_D03
87.2 mk12090_D03
87.3 mk12085_D03
87.5 mk12086_D03
87.6 mk12095_D03
87.8 mk12094_D03
89.1 mk12092_D03 mk12091_D03
mk12093_D03
90.1 mk12097_D03 mk12096_D03
94.5 mk12127_D03 mk12129_D03
94.6 mk12126_D03
95.1 mk12125_D03
95.4 mk12121_D03 mk12120_D03
mk12119_D03
95.7 mk12123_D03
96.0 mk12122_D03
96.1 mk12124_D03
99.2 mk12128_D03
102.0 mk12115_D03
103.2 mk12108_D03
103.9 mk12107_D03
105.0 mk12098_D03
105.8 mk12109_D03
106.5 mk12111_D03
106.7 mk12110_D03
107.6 mk12112_D03
108.6 mk12118_D03
109.6 mk12113_D03
109.7 mk12114_D03
110.0 mk12116_D03 mk12117_D03
113.5 mk12130_D03
113.7 mk12131_D03
114.7 mk12133_D03
114.8 mk12135_D03
115.0 mk12136_D03
116.0 mk12137_D03
116.2 mk12139_D03
116.3 mk12138_D03
120.7 mk12140_D03
124.3 mk12143_D03
125.1 mk12142_D03
134.6 mk12159_D03
135.0 mk12160_D03
141.7 mk12152_D03 mk12155_D03
142.2 mk12154_D03
143.6 mk12156_D03
143.8 mk12153_D03
145.3 mk12158_D03
150.2 mk12161_D03
153.4 mk12169_D03
153.8 mk12170_D03
154.5 mk12163_D03
154.6 mk12165_D03 mk12164_D03
154.7 mk12162_D03 mk12166_D03
156.3 mk20098 mk20097
156.9 mk12168_D03
157.2 mk12167_D03
157.6 mk12171_D03
161.1 mk19256
170.6 mk12186_D03
qFW_D03_ck***
qLFW_D03_ck.1
qLFW_D03_ck.2
qLFW_D03_ck.3
qSLW_D03_ck.1***
qSLW_D03_ck.2***
qSLW_D03_ck.3***
qDLW_D03_ck.1***
qDLW_D03_ck.2***
qDLW_D03_ck.3***
qFW_D03_110.1***
qFW_D03_110.2***
qSLW_D03_110***
qDLW_D03_110***
qMDA_D03_150.1
qMDA_D03_150.2
qFW_D03_150.1***
qFW_D03_150.2***
qSLW_D03_150***

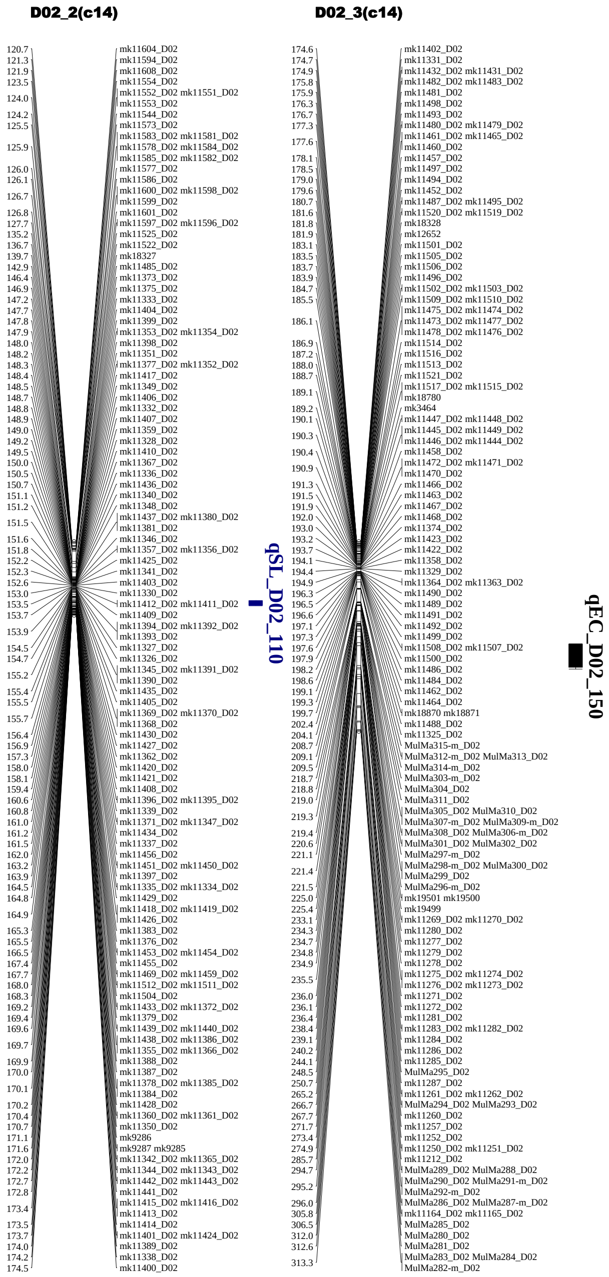
D02_2(c14)
D02_3(c14)
qSL_D02_110
qEC_D02_150

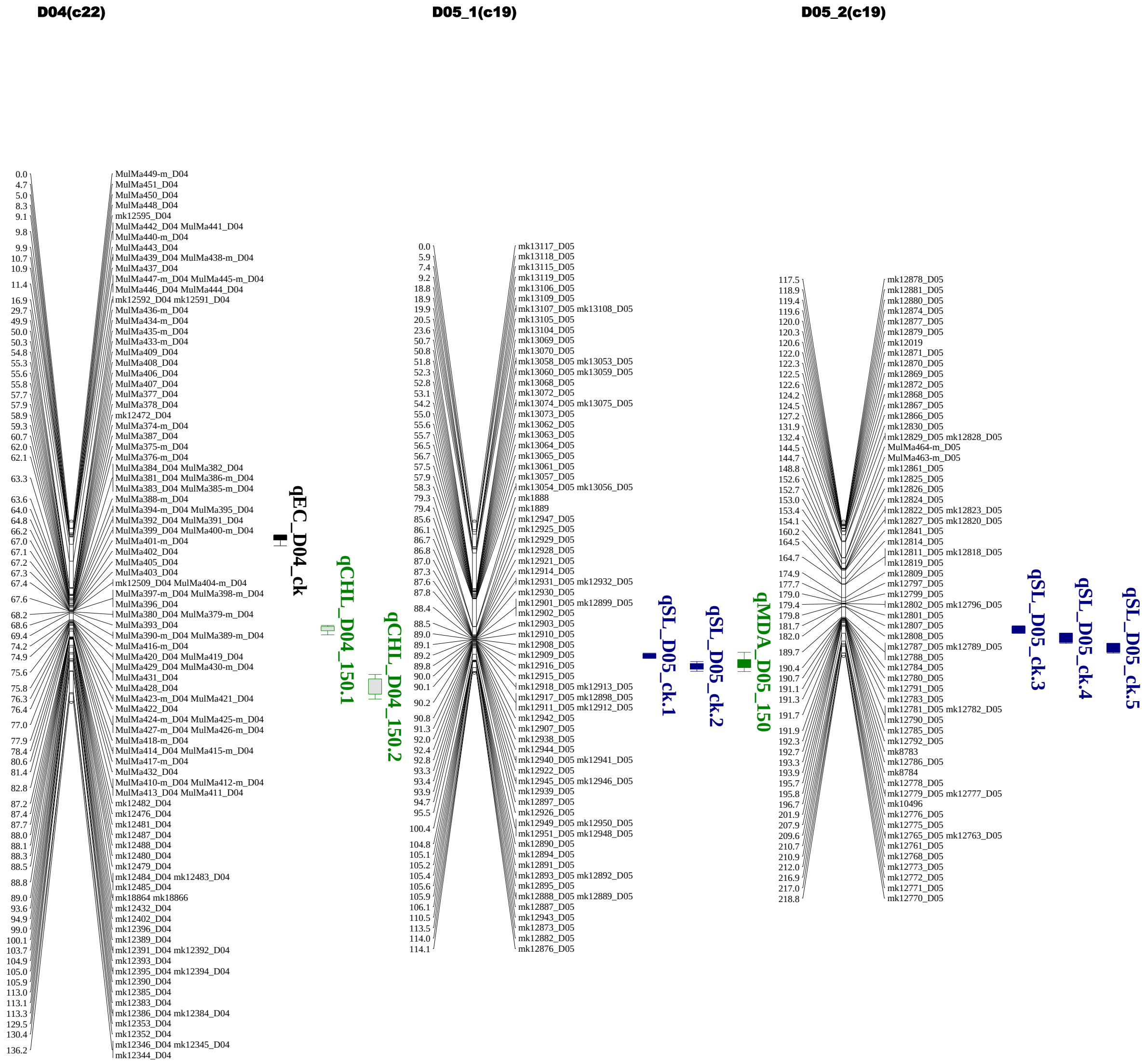

D04(c22)
D05_1(c19)
D05_2(c19)
qEC_D04_ck
qCHL_D04_150.1
qCHL_D04_150.2
qSL_D05_ck.1
qSL_D05_ck.2
qMDA_D05_150
qSL_D05_ck.3
qSL_D05_ck.4
qSL_D05_ck.5

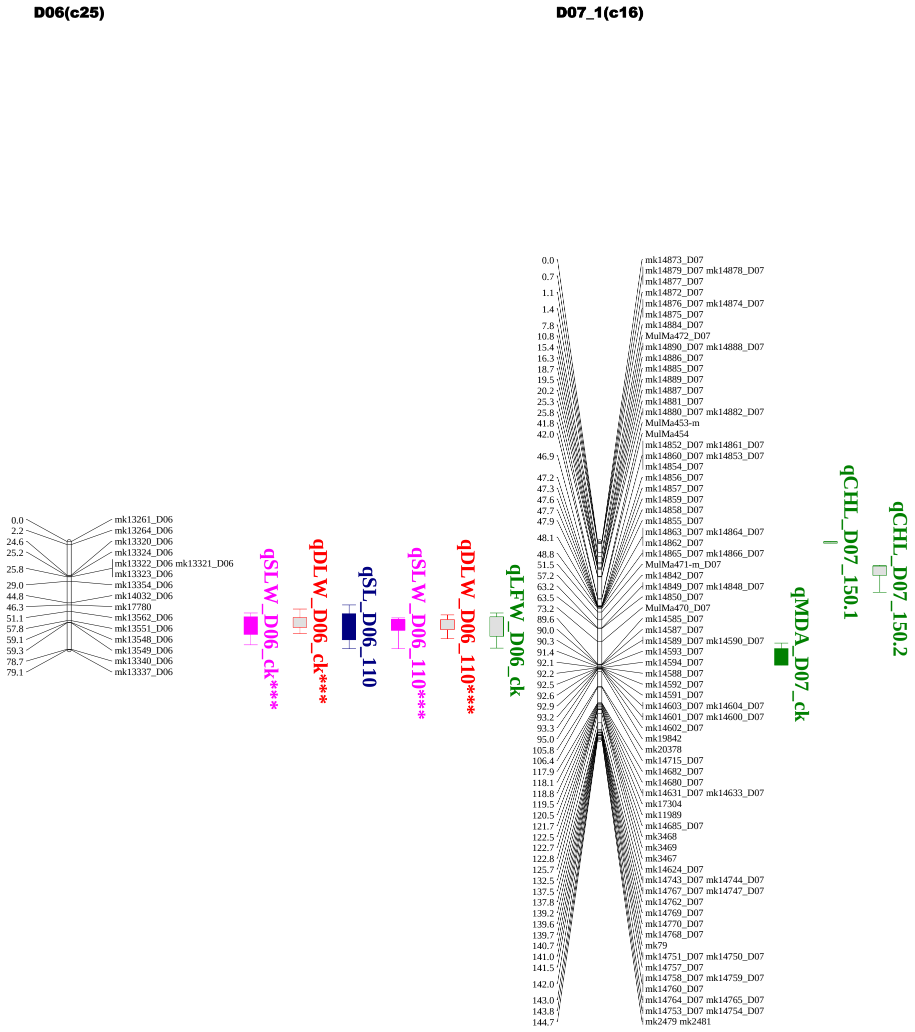

D06(c25)
D07_1(c16)
qSLW_D06_ck***
qDLW_D06_ck***
qSL_D06_110
qSLW_D06_110***
qDLW_D06_110***
qLFW_D06_ck
qMDA_D07_ck
qCHL_D07_150.1
qCHL_D07_150.2

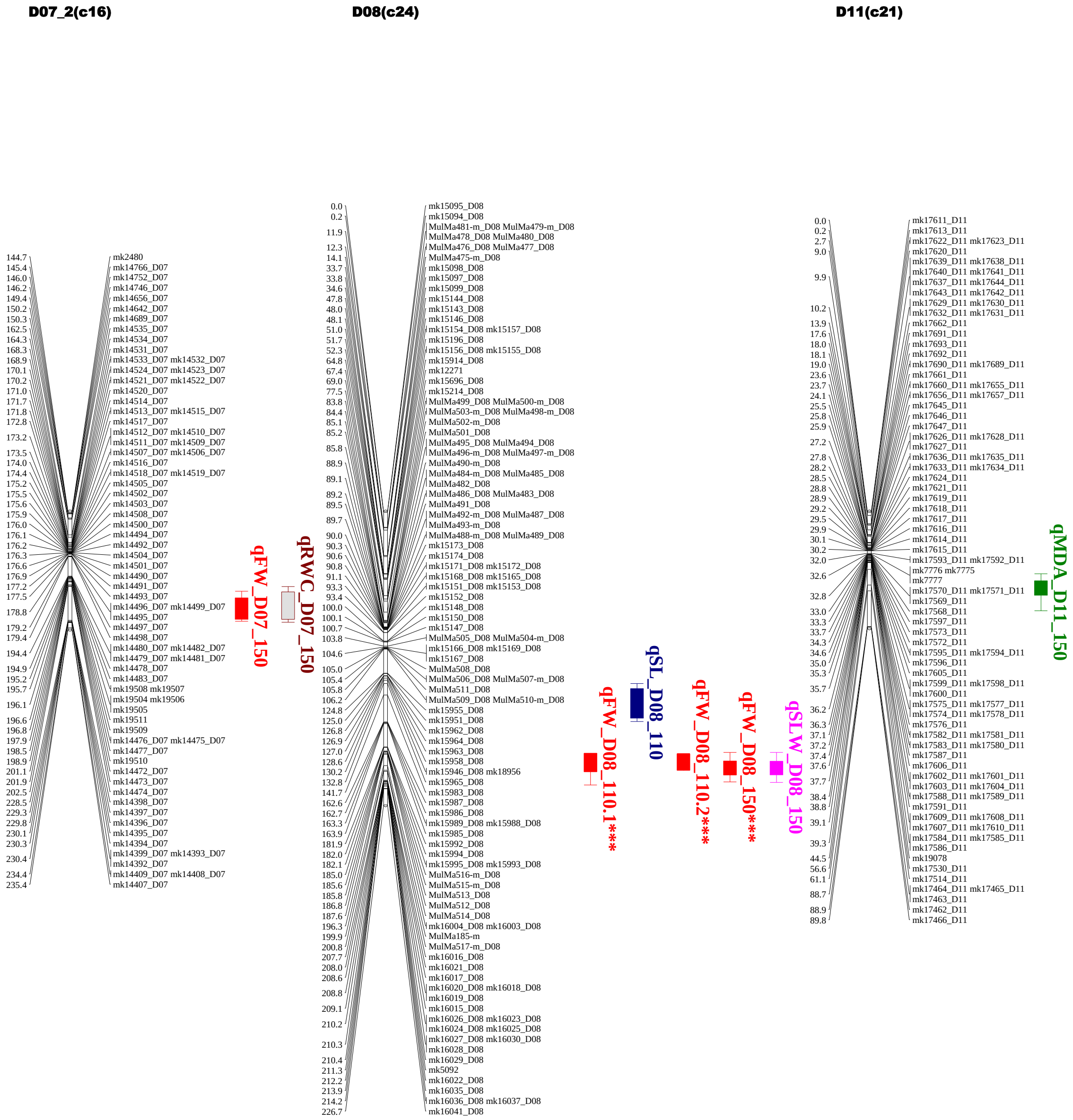

D07_2(c16)
D08(c24)
D11(c21)
qFW_D07_150
qRWC_D07_150
qSL_D08_110
qFW_D08_110.1***
qFW_D08_110.2***
qFW_D08_150***
qSLW_D08_150
qMDA_D11_150

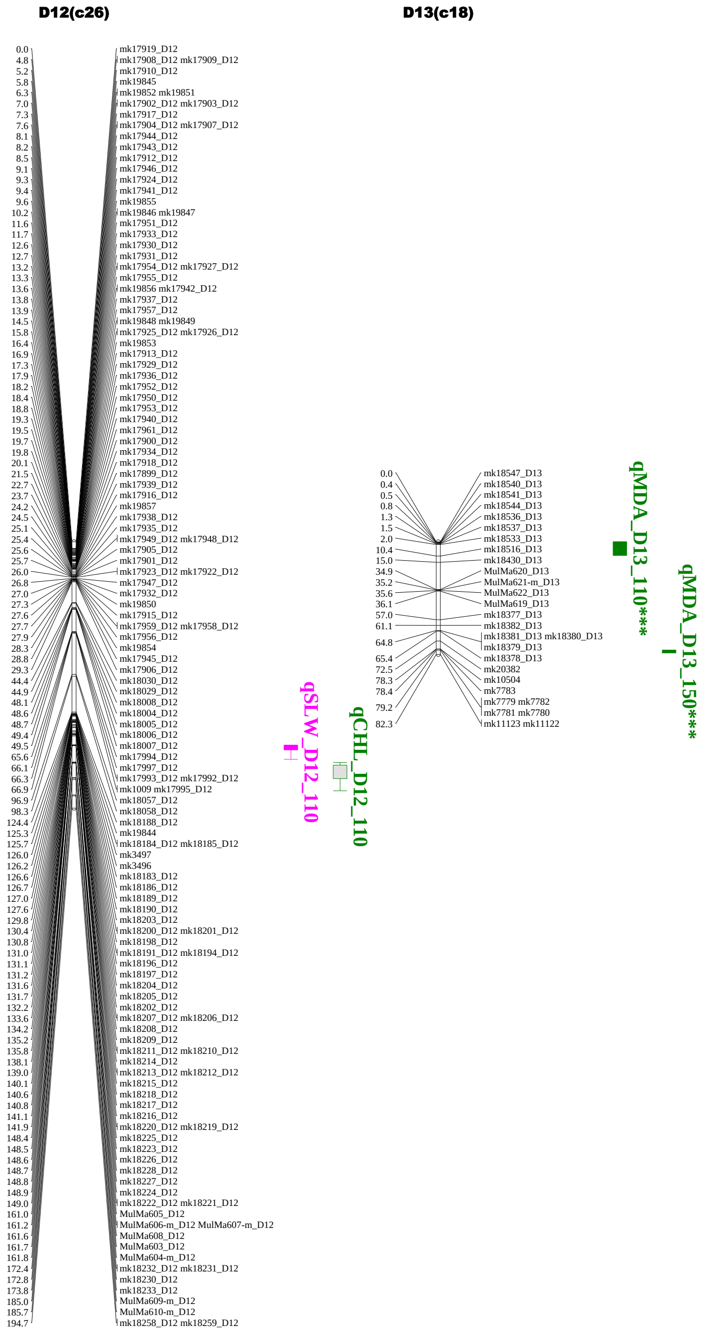

Figure S2: Total QTLs (66) found in this study, asterisk means consistent QTLs
